# TUBB2B facilitates progression of hepatocellular carcinoma by regulating cholesterol metabolism through targeting HNF4A/CYP27A1

**DOI:** 10.1038/s41419-023-05687-2

**Published:** 2023-03-06

**Authors:** Xiaobo Wang, Jiawei Shi, Mingming Huang, Jiehong Chen, Jia Dan, Yunhua Tang, Zhiyong Guo, Xiaoshun He, Qiang Zhao

**Affiliations:** 1 Department of Spinal Surgery, Nanfang Hospital, Southern Medical University, Guangzhou, China; 2 Organ Transplant Center, the First Affiliated Hospital, Sun Yat-Sen University, Guangzhou, China; 3 Guangdong Provincial Key Laboratory of Organ Donation and Transplant Immunology, Guangzhou, China; 4 Guangdong Provincial International Cooperation Base of Science and Technology, Guangzhou, China; 5 Department of Pharmacology, Zhongshan School of Medicine, Sun Yat-sen University, Guangzhou, China

**Keywords:** Cancer metabolism, Tumour biomarkers

## Abstract

Cholesterol metabolism plays a critical role in the progression of hepatocellular carcinoma (HCC), but it is not clear how cholesterol metabolism is regulated. The tubulin beta class I genes (TUBBs) are associated with the prognosis of many different cancers. To confirm the function of TUBBs in HCC, the Kaplan–Meier method and Cox analyses were performed using TCGA and GSE14520 datasets. A higher expression of TUBB2B is an independent prognostic factor for shorter over survival in HCC patients. Deletion of TUBB2B in hepatocytes inhibits proliferation and promotes tumor cell apoptosis, while over-expression of TUBB2B has the opposite function. This result was confirmed in a mouse xenograft tumor model. Mechanistically, TUBB2B induces the expression of CYP27A1, an enzyme responsible for the conversion of cholesterol to 27-hydroxycholesterol, which leads to the up-regulation of cholesterol and the progression of HCC. In addition, TUBB2B regulates CYP27A1 via human hepatocyte nuclear factor 4alpha (HNF4A). These findings indicated that TUBB2B functions as an oncogene in HCC, and plays a role in promoting cell proliferation and anti-apoptosis through targeting HNF4A/CYP27A1/cholesterol.

## Introduction

Hepatocellular carcinoma (HCC) is one of the most prevalent malignancies, and results in more than 700,000 deaths worldwide every year [1]. Although the treatment such as tumor ablation, resection, chemoembolization, and liver transplantation have substantially improved the prognosis for patients with HCC over the past decade [2], patients with HCC are usually diagnosed with a poor prognosis due to high recurrence and metastasis [3]. Thus, it is important to further elucidate the molecular mechanism of HCC in order to develop more efficient treatment.

The tubulin beta class I genes (TUBBs) encode several beta tubulin proteins that are essential components of microtubules. TUBBs are related to growth, infiltration, and drug resistance in several different malignancies [4]. For example, mutation of TUBBs impacts taxane resistance in breast cancer [5], and higher expression of TUBBs is correlated with worse survival in non-small cell lung cancer (NSCLC) by regulating chemoresistance [5, 6]. Moreover, TUBBs are involved in the carcinogenesis and progression of NSCLC and pancreatic cancer [7]. However, little is known about the function of TUBB family members in HCC.

Alteration of lipid metabolism is considered to be one of the hallmarks of cancer, and a key event during tumor initiation and progression [8]. Epidemiological studies have indicated that cholesterol intake is an independent risk factor for HCC [9]. Cholesterol is primarily synthesized in the liver and is an essential lipid for maintaining cellular homeostasis [10]. Dysregulation of cholesterol metabolism is frequently observed in HCC [11], and activation of cholesterol synthesis drives tumorigenesis [12]. Thus, reducing cholesterol level via inhibiting its biosynthesis or promoting its degradation may be an effective strategy to suppress HCC progression.

In this study, we identified the prognostic value of TUBBs in HCC patients. Then we confirmed that higher TUBB2B expression is associated with a poor prognosis in HCC patients, and that sh-TUBB2B inhibits proliferation and promote apoptosis in human HCCs. We also found that TUBB2B modulates cholesterol metabolism via targeting CYP27A1, an enzyme responsible for the conversion of cholesterol to 27-hydroxycholesterol, to promote tumor progression. Finally, we showed that TUBB2B regulates CYP27A1 via human hepatocyte nuclear factor 4alpha (HNF4A). These findings may provide the basis for new targeted treatments for HCC.

## Materials and methods

### Patients and HCC tissue samples

Clinical information and transcriptomic data of HCC samples were downloaded from The Cancer Genome Atlas (TCGA) data portal (https://portal.gdc.cancer.gov/), and from the Gene Expression Omnibus (GEO) (https://www.ncbi.nlm.nih.gov/gds/) for bioinformatic analysis. For this study, the data were referred to as entire TCGA cohort (*n* = 365) and the GSE14520-GPL3921 (GSE14520) cohort (*n* = 221), respectively. For validation, tumor tissues and matched normal tissues at 6 cm from the tumor margin were collected from 74 patients with HCC who received curative surgery at the First Affiliated Hospital, Sun Yat-sen University. The samples were stored in liquid nitrogen until use, and tested for TUBB2B by quantitative real-time PCR (RT-PCR). This research was approved by the Ethical Review Committee. Written informed consent was received according to the guidelines of the Declaration of Helsinki.

### RNA extraction and quantitative RT-PCR

Total RNA was extracted from HCC cell lines and fresh tumor tissues using TRIzol Reagent (Thermo Scientific, USA) according to the manufacturer’s protocol. A total of 2 μg RNA was reverse-transcribed to cDNA with Oligo(dT) (synthesized by Life Technologies), and RevertAid Reverse Transcriptase (Thermo Scientific). Expression of specific genes was calculated by the comparative cycle threshold method using SuperReal PreMix SYBR Green (FP204-02; TIANGEN) in an Applied Biosystem 7500 Fast RT-PCR system (Life Technologies). Primer sequences are shown in the Supplementary material.

### Cell culture and reagents

Cell lines were purchased from American Type Culture Collection and Shanghai Institute of Cell Biology, and none of the cells used are listed in the database of commonly misidentified cell lines maintained by the International Cell Line Authentication Committee (Version 8.0). All cell lines were authenticated by the short tandem repeat (STR) assay, and were confirmed to be without mycoplasma contamination. Cells were cultured in Dulbecco’s modified Eagle’s medium (DMEM, Gibco, USA) supplemented with 10% (vol/vol) fetal bovine serum (FBS) and 1% penicillin/streptomycin. Cells were cultured in a humidified atmosphere with 5% CO_2_ at 37 °C. Exponentially growing cells were used for the subsequent experiments.

### Knock-down and over-expression in HCC cell lines

Cells were plated at a density of 200,000 cells per well of a six-well plate, 24 h prior to transfection. Transfection of Hep3B and Huh7 cells was performed using Lipofectamine™ 2000 (Thermo Fisher Scientific), in serum- and antibiotic-free OptiMem™ I Reduced Serum medium (Thermo Fisher Scientific), according to manufacturer’s instructions. Plasmids containing short hairpin RNA (shRNA) molecules directed against human TUBB2B, HNF4A, and CYP27A1 mRNA, or a scrambled shRNA (Thermo Fisher Scientific) were used. At 24, 48 and 72 h post-transfection, the cells were collected for Western blot analysis. Sequences of the shRNA used are shown in Supplementary file 3.

### Western blotting

Total protein was extracted from tissues or cells using ice-cold radioimmunoprecipitation assay (RIPA) buffer containing protease and phosphatase inhibitors (Cell Signaling Technology, USA). Protein samples were separated using sodium dodecyl sulfate-polyacrylamide gel electrophoresis (SDS-PAGE), transferred to polyvinylidene fluoride (PVDF) membranes (Merck Millipore, Burlington, MA, USA), and blocked with 5% skim milk in Tris-buffered saline containing Tween-20 (TBST) at room temperature for 1 h. Membranes were probed with primary antibodies at 4 °C overnight with gentle rocking, followed by incubation with horseradish peroxidase (HRP)-conjugated secondary antibodies for 1 h at room temperature before visualization using ECL kits (Thermo Scientific, USA). The membranes were visualized with a ChemiDoc XRS + System (Bio-Rad) using Immobilon Western Chemiluminescent HRP Substrate (Millipore). Full and uncropped western blots are in Supplemental Material 3.

### Cell counting kit-8 (CCK8) assay

Cells were seeded into 48-well culture plates (1 × 10^4^ cells/well). After 12 h of cultivation, the cells were transfected with siRNAs. CCK-8 solution (30 μL, Dojindo, Kumamoto, Japan) was added to each well at different time points, and the cells were cultured of an additional for 1.5 h. Samples’ optical density (OD) at 450 nm was then measured.

### Cell-light 5-ethynyl-2-deoxyuridine (EdU) assay

The EdU Apollo567 In Vitro kit (Ribobio, China) was used to assess cell proliferation. Cells transfected with miRNA mimics for 24 h were seeded into 6-well plates (2 × 10^5^ cells/well). After culture for 12 h cultivated, the cells were transfected for 24 h and were cultivated for 40 h. The cells were then incubated in EdU working solution for 2 h, fixed with 4% paraformaldehyde, permeabilized, washed, and stained with 1× Apollo solution and 1× Hoechst33342 solution, according to the manufacturer’s instructions. Results were analyzed from microphotographs taken using a fluorescence microscope.

### Xenograft studies in nude mice

Four-week-old male BALB/c nude mice were purchased from Shanghai Experimental Animal Center of the Chinese Academic of Sciences (Shanghai, China). A total of 4 × 10^6^ Huh7 and Hep3B cells suspended in 100 μl PBS were subcutaneously injected into the flanks of the mice (4 mice per group, randomly selected). Four weeks after injection, the mice were killed and the tumors were surgically dissected and collected. All animal experiments were approved by the Ethics Committee for Laboratory Animals of the First Affiliated Hospital, Sun Yat-sen University.

### Cholesterol concentration determination

Total cholesterol determination was carried out as previously reported [13]. Briefly, 10 mg of cell lysate was saponified with alcoholic KOH in a 60 °C heating block for 30 min. Then 3 ml of hexane and 600 μl of distilled water were added and the mixture was shaken to ensure complete mixing. After evaporation, cholesterol was detected with O-phthalaldehyde dissolved in acetic acid (0.5 mg/ml). Then, sulfuric acid (1 ml) was added, and then the OD at 550 nm was read using a spectrophotometer.

### Statistical analysis

Statistical analyses were performed using SPSS version 22.0 software (SPSS, Chicago, IL, USA), Prism 7.0 software (GraphPad Software, La Jolla, CA, USA) and R language version 3.6.1. Data were presented as the mean ± standard deviation of at least three independent experiments. Quantitative data were compared using a two-sided Student’s t-test or Wilcoxon matched-pairs test. Categorical data compared with Fisher exact test. Over survival (OS) was evaluated by the Kaplan–Meier (K-M) survival curve and the log-rank test. Prognostic factors were estimated using a univariate Cox proportional hazards regression model and a multivariate Cox model. A two-tailed *p* < 0.05 was considered statistically significant.

## Results

### Expression and prognostic value of TUBBs in patients with HCC

**Fig. 1:**
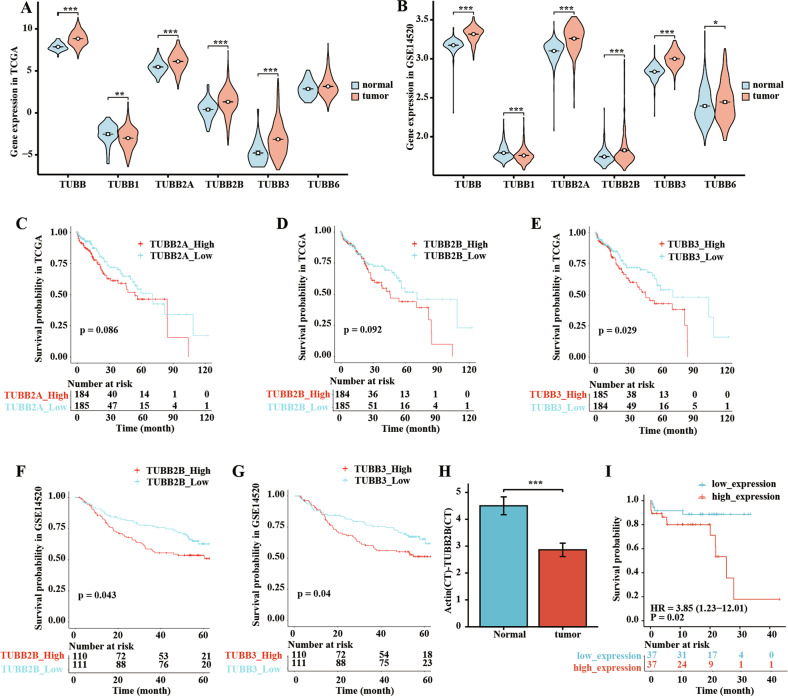
Expression and prognostic value of TUBBs in HCC. **A**, **B** The expression levels of TUBB family genes in HCC patients from TCGA and GSE14520 databases. **C**–**G** Kaplan–Meier curves show the impact of the genes on survival outcomes from TCGA and GSE14520 databases. **H** Quantitative real-time PCR analysis indicated significantly increased TUBB2B expression in HCC tissue compared with matched normal tissue in 74 HCC patients. **I** Kaplan–Meier survival curves comparing the outcome of 74 HCC patients stratified by TUBB2B expression. *p < 0.05, **p < 0.01, ***p < 0.001.

To determine the expression of TUBBs in HCC, we compared data between normal tissue and tumor tissue using TCGA and GSE14520 databases. We found that TUBB, TUBB2A, TUBB2B, and TUBB3 exhibited higher expression in HCC tissues than normal tissues in both databases (Fig. 1A, B). Next, the K-M method was used to determine the prognostic values of the expressions of TUBBs. As shown in Fig. 1C–E, analysis revealed that the higher expression levels of TUBB2A, TUBB2B, and TUBB3 were associated with shorter OS (*p* < 0.05) in the TCGA HCC cohort, while higher mRNA levels of TUBB2B and TUBB3 were associated with shorter OS in the GSE14520 cohort (*p* < 0.05) (Fig. 1F, G). Others TUBBs did not exhibit significant differences in prognosis when stratified by expression level (Fig. S1A–G).

To assess the independent prognostic value of TUBBs expression in HCC patients, we conducted Cox survival regression analysis. Patient clinical characteristics are summarized in Supplementary Tables 4 and 5. Univariate analysis showed that TUBB2B was significantly related to OS in both TCGA (hazard ratio [HR] = 1.06, 95% confidence interval [CI]: 1.02–1.10, and *p* = 0.004) and GSE14520 HCC patients (HR = 1.36, 95% CI: 1.10–1.69, and *p* = 0.005) (Supplementary Tables 6 and 7). Multivariate analysis showed similar results for TCGA (HR = 1.05, 95% CI: 1.00–1.09, and *p* = 0.039) (Supplementary Table 6) and GSE14520 HCC patients (HR = 1.40, 95% CI: 1.09–1.79, and *p* = 0.009) (Supplementary Table 7).

The discrepancies of the results for expression and prognostic value of TUBBs between TCGA and GSE14520 datasets are likely due to varying analysis platforms analyzing different gene sets. To address this, we merged all data by taking the intersection of the two databases, and analyzed TUBB2B expression in 74 HCC patients. Then results showed that the expression of TUBB2B was higher in HCC tissue than paired normal tissue (Fig. 1H). K-M analysis also showed that higher TUBB2B expression was associated with shorter OS (Fig. 1I). Taken together, these results indicated that high expression of TUBB2B may be a reliable indicator of a poor prognostic for patients with HCC.

### TUBB2B under-expression suppresses tumor proliferation and promoted tumor cell apoptosis in vitro and in vivo

**Fig. 2:**
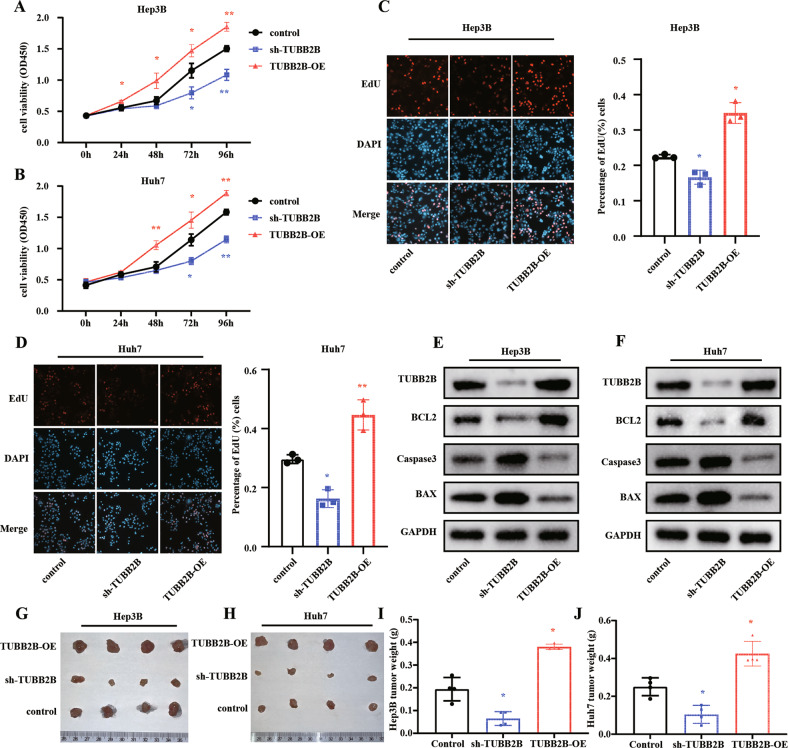
TUBB2B deficiency suppresses HCC proliferation, and promotes apoptosis in vitro and in vivo. **A**–**D** The function of TUBB2B on cell proliferation was determined by CCK8 assay and EdU quantification in Hep3B and Huh7 cells (*n* = 3/per group). **E**, **F** Effects of TUBB2B on apoptosis biomarkers (*n* = 3/per group). **G**, **H** Image of Hep3B and Huh7 xenografted mice sacrificed at 1 month (*n* = 4/per group). **I**, **J** Tumor weight in control, sh-TUBB2B, and TUBB2B-OE groups are shown (*n* = 4/per group). Experimental group compared to control group: *p < 0.05, **p < 0.01.

We examined the function of TUBB2B in Huh7 and Hep3B cell lines by knock-down and over-expression of TUBB2B (Fig. S2A, B). CCK-8 assay results indicated that TUBB2B deficiency decreased cell viability in Huh7 cells (Fig. 2A) and Hep3B cells (Fig. 2B) (both, *p* < 0.05), while TUBB2B overexpression increased cell viability. The EdU assay results suggested that sh-TUBB2B inhibited cell proliferation while TUBB2B-OE increased cell proliferation (Fig. 2C, D). In addition, sh-TUBB2B significantly increased apoptosis and TUBB2B-OE significantly decreased apoptosis, which studied by the detection of BCL2, BAX, and Caspase3 (Fig. 2E, F).

To confirm the role of TUBB2B in vivo, a tumor xenograft models was constructed by subcutaneously injecting HCC cells with either sh-TUBB2B or TUBB2B-OE into nude mice. As shown in Fig. 2G, H, TUBB2B knock-down significantly reduced tumor growth rate, while over-expression TUBB2B increased the tumor growth rate resulting in bigger and heavier tumors.

### CYP27A1 is a potential target of TUBB2B in HCC

**Fig. 3:**
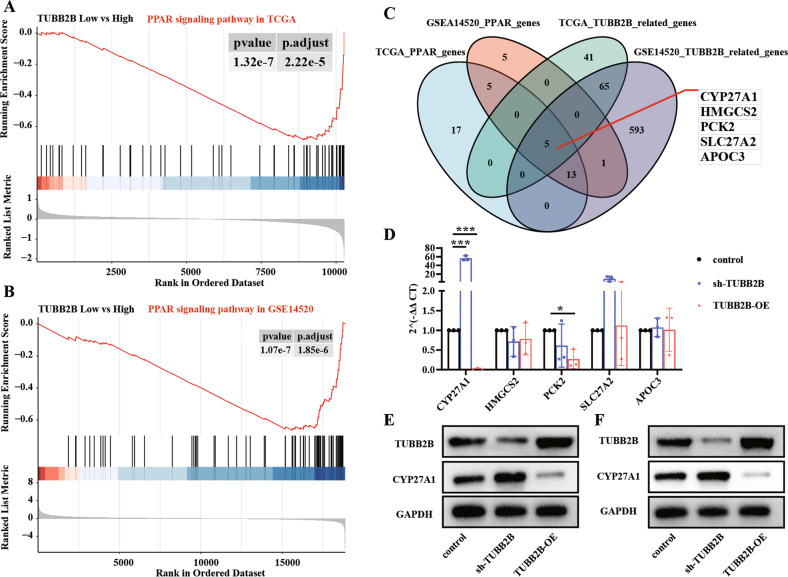
CYP27A1 is a target of TUBB2B. **A**, **B** GSEA enrichment plot of the KEGG PPAR signaling pathway in TCGA and GSE14520 databases stratified by TUBB2B expression (high ≥ median expression, low < median expression). **C** Venn diagrams for genes that are enriched in the PPAR pathway, and genes significantly related to TUBB2B expression in TCGA and GSE14520 databases. **D** Quantitative real-time PCR showing that CYP27A1 is upregulated after knock-down of TUBB2B and is downregulated after over-expression of TUBB2B in Hep3B (*n* = 3/per group). **E**, **F** Western blot analysis of CYP27A1 protein level in Hep3B and Huh7 cells after treatment with sh-TUBB2B or TUBB2B-OE. *p < 0.05, ***p < 0.001.

To determine the mechanism by which TUBB2B affects HCC, data from TCGA and GSE14520 were divided into a high-expression group and a low-expression group based on median TUBB2B expression (high ≥ median expression, low < median expression). Differential expression gene analysis and gene set enrichment analysis (GSEA) of KEGG pathways were performed. The GSEA analysis revealed that most of the enriched pathways were correlated with lipid metabolism (Fig. S2C, D), suggesting that TUBB2B expression may affect lipid metabolism in tumor development. In addition, the peroxisome proliferator-activated receptor (PPAR) pathway, which is essential for lipid metabolism [14], was significantly downregulated in the TUBB2B high-expression groups (Fig. 3A, B).

Next, we assessed the role of TUBB2B in the PPAR signaling pathway. The PPAR-related genes enriched in the high- and low-expression TUBB2B group, and the correlation between TUBB2B expression and other enriched genes in the two datasets were studied (threshold: correlation coefficient < −0.15 and *p* < 0.01). The intersection of the four results was identified (Fig. 3C). Five PPAR-related genes, CYP27A1, HMGCS2, PCK2, SLC27A2, and APOC3 were closely associated with TUBB2B (Fig. 3C). Quantitative RT-PCR was performed to assess the impact of TUBB2B in on each candidate, and the results showed that silencing TUBB2B significantly upregulated CYP27A1 expression, while TUBB2B over-expression downregulated CYP27A1 (Fig. 3D). The Western blot analysis validated the results in Huh7 and Hep3B cell lines (Fig. 3E, F).

In addition, the expression of CYP27A1 was lower in HCC tumor than adjacent normal tissue (Fig. S3A, B), and lower expression of CYP27A1 in tumor tissue was associated with worse OS in TCGA and GSE14520 cohorts (Fig. S3C, D).

### CYP27A1 promotes HCC progression through modulating cholesterol degradation

**Fig. 4:**
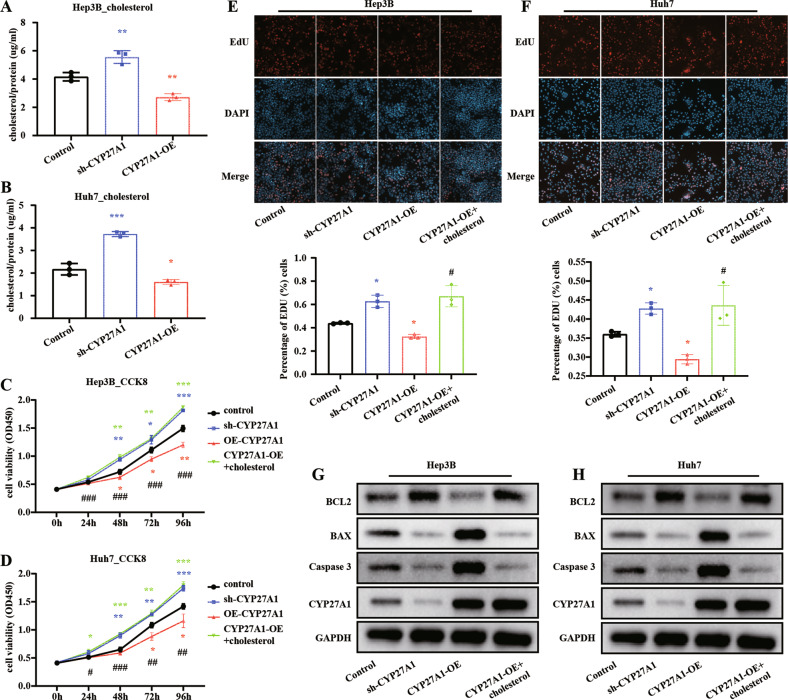
CYP27A1 promotes HCC progression through modulating cholesterol degradation. **A**, **B** Cholesterol levels in Hep3B and Huh7 cells treated as shown. **C**–**F** Cell proliferation of Hep3B and Huh7 cells measured by CCK8 and EdU assays. Quantification of EdU results after indicated treatment. **G**, **H** Western blot analysis of apoptotic markers after different treatments of Hep3B and Huh7 cells. *n* = 3/per group. Experimental group compared to control group: *p < 0.05, **p < 0.01, ***p < 0.001. CYP27A1-OE group compared to CYP27A1-OE + cholesterol group: #*p* < 0.05, ##*p* < 0.01, ###*p* < 0.001.

As CYP27A1 is an important enzyme involved in regulating cellular cholesterol homeostasis by converting cholesterol to 27-hydroxycholesterol [15], we considered that CYP27A1 might mediate HCC progression by regulating cholesterol metabolism. As expected, sh-CYP27A1 resulted in an increase in cholesterol level in both HCC cell lines, while CYP27A1-OE decreased cholesterol level in both HCC cell lines (Fig. 4A, B). Moreover, CYP27A1-OE cell viability (Fig. 4C, D) and proliferation (Fig. 4E, F) in Huh7 cells and Hep3B cells, while sh-CYP27A1 overexpression increased cell viability and proliferation (all, *p* < 0.05). CYP27A1-OE significantly increased apoptosis and sh-CYP27A1 significantly decreased apoptosis (Fig. 4G, H). But exogenous cholesterol could counteract the effect of CYP27A1-OE on cell viability, proliferation, and the levels of apoptosis markers (BCL2, BAX, and Caspase3) (Fig. 4C–H). Taken together, these results indicated that CYP27A1 inhibits HCC progression by lowering cholesterol level.

### TUBB2B regulates CYP27A1 to affect cholesterol degradation, cell proliferation, and apoptosis

**Fig. 5:**
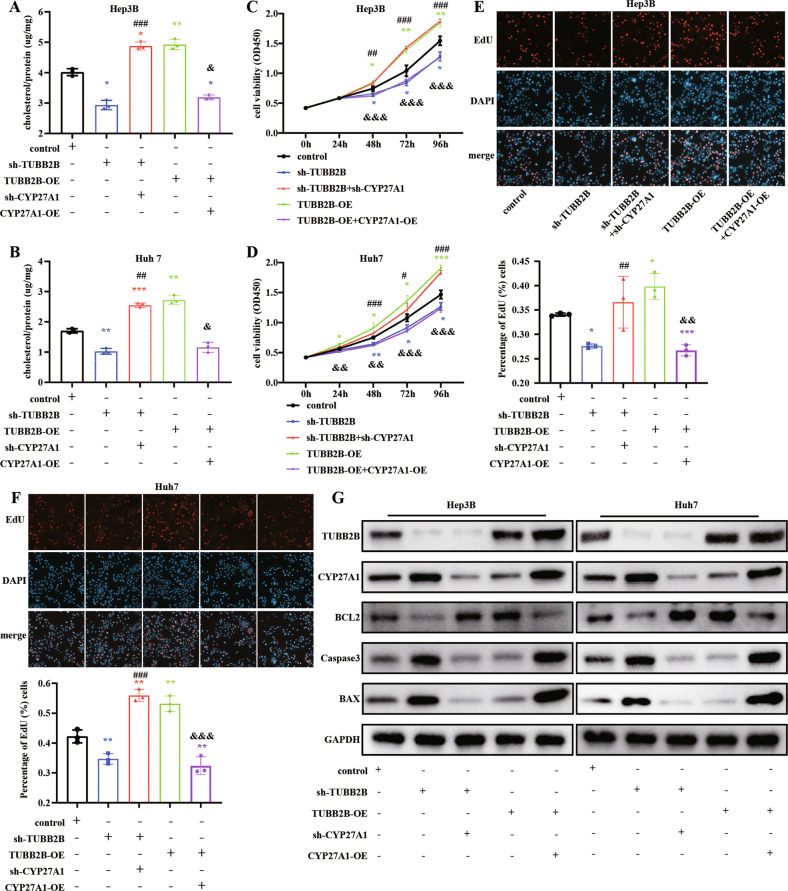
TUBB2B regulates CYP27A1 to affect cholesterol degradation and cell proliferation and apoptosis. **A**, **B** Cholesterol level in Hep3B and Huh7 cells treated as shown. **C**–**F** Cell proliferation of Hep3B and Huh7 cells measured by CCK8 and EdU assays. Quantification of EdU results after indicated treatment. **G** Western blot analysis of apoptotic markers in Hep3B and Huh7 cells after different treatment (*n* = 3/per group). Experimental group compared to control group: *p < 0.05, **p < 0.01, ***p < 0.001. sh-TUBB2B group compared to sh-TUBB2B + sh-CYP27A1 group: #*p* < 0.05, ##*p* < 0.01, ###*p* < 0.001. TUBB2B-OE group compared to TUBB2B-OE + CYP27A1-OE: &*p* < 0.05, &&*p* < 0.01, &&&*p* < 0.001.

Based on the prior results, we determined if TUBB2B regulates cholesterol metabolism, cell proliferation, and apoptosis through affecting CYP27A1. Cholesterol levels were increased by TUBB2B-OE and decreased by sh-TUBB2B, and this effect was reversed by sh-CYP27A1 or CYP27A1-OE in Huh7 cells and Hep3B cells (Fig. 5A, B). CCK-8 assay showed that CYP27A1 knock-down reversed the effect of sh-TUBB2B on cell viability, while CYP27A1 over-expression reversed the suppressive effect of TUBB2B-OE on cell viability (Fig. 5C, D). Finally, knock-down of CYP27A1 reversed the effect of sh-TUBB2B on cell proliferation and apoptosis (detected by apoptotic biomarkers, BCL2, BAX, and Caspase3), and over-expression of CYP27A1 reversed the effect of TUBB2B-OE on cell proliferation and apoptosis (Fig. 5E–G).

### TUBB2B suppresses CYP27A1 and cholesterol degradation by regulating HNF4A

**Fig. 6:**
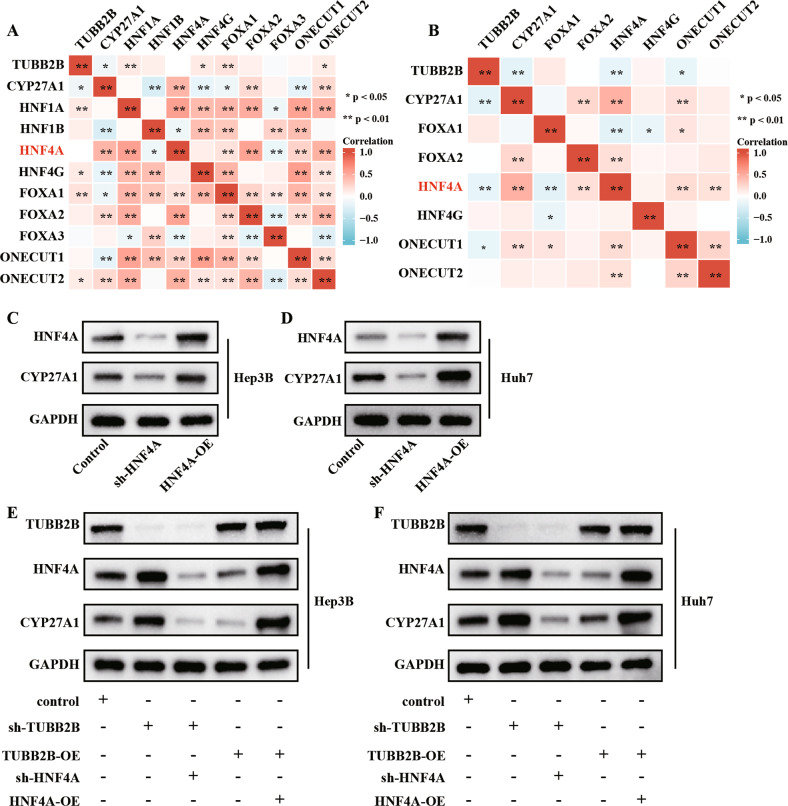
TUBB2B suppression of CYP27A1 and cholesterol degradation is mediated by HNF4A. **A**, **B** Heatmap showing the correlation of TUBB2B, CYP27A1, and hepatocyte nuclear factors from TCGA and GSE14520 data. **C**, **D** Representative Western blot analysis of HNF4A and CYP27A1 after treatment with sh-HNF4A or HNF4A-OE. **E**, **F** Representative Western blot analysis of TUBB2B, HNF4A, and CYP27A1 after indicated treatment.

Hepatocyte nuclear factors (HNFs) are transcription factors that have been demonstrated to regulate CYP27A1 [16, 17]. The HNF family includes HNF1A/B, FOXA1/2/3, HNF4A/G, and ONECUT1/2. To understand whether TUBB2B regulated CYP27A1 via HNFs, we explored the association of TUBB2B and CYP27A1 with HNFs. The results showed that HNF4A was negatively related to TUBB2B expression, and positively related to CYP27A1 expression in TCGA and GSE14520 cohorts, while other HNFs exhibited no relations (Fig. 6A, B).

Therefore, we hypothesized that TUBB2B may regulate CYP27A1 through HNF4A. Knock-down of HNF4A decreased the expression of CYP27A1, while over-expression of HNF4A increased the expression of CYP27A1 (Fig. 6C, D). In both HCC cell lines, sh-TUBB2B caused an increase in HNF4A expression, TUBB2B-OE decreased HNF4A expression (Fig. 6E, F). Furthermore, knock-down of HNF4A reversed the effect of sh-TUBB2B on CYP27A1, and HNF4A over-expression reversed the suppressive effect of TUBB2B-OE on CYP27A1 (Fig. 6E, F).

## Discussion

Our study demonstrated that TUBB2B plays a role in promoting HCC progression. Over-expression of TUBB2B promotes HCC growth and reduces apoptosis by increasing cholesterol level via inhibiting CYP27A1. Furthermore, over-expression of TUBB2B increases the expression of CYP27A1 through HNF4A in HCC cell lines. Thus, TUBB2B might be a potential target for decreasing cholesterol degradation in HCC.

TUBBs encode dimeric proteins that are the major components of microtubules, their roles have been primarily studied in cortical development. Currently, evidence is accumulating that they play a role in the progression of malignancies. A quantitative comparison of tubulin isotype protein levels showed that TUBBs predominate among tubulin family member in normal and breast cancer tissue [18], and all TUBBs significantly increased in paclitaxel-resistant lung cancer cells [19]. TUBB2B is highly expressed and associated with poor OS in endometrial cancer [20] and neuroblastoma [21]. Consistently, by analyzing expression and prognostic value of TUBBs in TCGA and GSE14520 data, we found that TUBB2B expression was higher in HCC tissue than adjacent normal tissue and expression was correlated with the HCC prognosis. These results were confirmed in tissue samples from 74 patients with HCC. Gain-of-function experiments demonstrated that over-expression of TUBB2B promoted proliferation and inhibited apoptosis in Hep3B and Huh7 cells, and knock-down of TUBB2B resulted in the opposite results. A xenograft murine model confirmed that TUBB2B played a functional regulatory role in the pathogenesis of HCC. Our findings suggest the promising prospect of in vivo delivery of siRNA targeting TUBB2B to promote OS for patients with HCC.

Cholesterol is an essential lipid component of cell membranes, which assists maintaining the integrity and fluidity of the membrane, and can form membrane microstructures [22]. Preclinical studies support that cholesterol homeostasis can modulate tumor development [23]. A recent study showed that high serum cholesterol levels reduced the growth of liver tumors in mice [24]. However, Ioannou et al. believed that cholesterol consumption was associated with a higher risk of cirrhosis or liver cancer, whereas serum cholesterol level was not associated with a risk of cirrhosis or liver cancer [9]. Montero et al. thought that mitochondrial cholesterol contributes to increase chemotherapy resistance in HCC [25]. In addition, several studies have demonstrated TUBBs have a capacity of regulating lipid metabolism. In mice fed a high-fat diet, the expression of TUBB2A and TUBB6 were significantly increased [26]. In humans, TUBB was reported to increase the risk of dyslipidemia and predict hypertension [27]. In the present study, we found that TUBB2B increased the cholesterol level in HCC cell lines, leading to an increase of proliferation and decrease of apoptosis.

A primary metabolite of cholesterol is 27-hydroxycholesterol, and CYP27A1 is the enzyme responsible for conversion of cholesterol to 27-hydroxycholesterol [28]. A study found that higher expression of CYP27A1 is associated with a higher grade of breast cancer and lower circulating cholesterol levels, but these changes were not related to prognosis [29]. Inhibiting cholesterol conversion to 27-hydroxycholesterol via CYP27A1 has been suggested to prevent breast cancer tumor progression [29]. However, a study of estrogen receptor positive primary breast cancer showed that high CYP27A1 expression is associated with favorable prognosis [30]. Studies demonstrated that CYP27A1 deficiency increased the proliferative activity of melanoma cells [31] and prostate cancer cells [32]. The present study found that CYP27A1 has a similar function in HCC. In addition, we found that TUBB2B plays an important role in the control of lipid metabolism, cell growth, proliferation, and apoptosis through regulating CYP27A1. Finally, we determine that HNF4A inhibits HCC by regulating CYP27A1, and TUBB2B regulates CYP27A1 through HNF4A. Other studies also demonstrated that HNF4A is an important transcription factor in hepatic lipid metabolism [33, 34], and HNF4A achieves this function by stimulating CYP27A1 gene transcription [35].

In conclusion, the current study identified an HCC-promoting effect of TUBB2B in patients, cell lines, and in vivo. TUBB2B functions by promoting CYP27A1 expression through facilitating HNF4A expression, which results in cholesterol degradation. The results suggest that a novel treatment for HCC may be targeting cholesterol degradation via TUBB2B/HNF4A/CYP27A1.

## Supplementary information

Supplemental file 1. Supplemental Figure S1-3.

Supplemental file 2. Supplemental Table 1-7

Supplemental file 3. Full and uncropped Western blots

Supplemental file 4. Reproducibility checklist

## Data Availability

The datasets used and/or analyzed during the current study are available from the corresponding author upon reasonable request.
