# Supplementary material for: TUBB2B facilitates progression of hepatocellular carcinoma by regulating cholesterol metabolism through targeting HNF4A/CYP27A1
Source: Cell Death Dis. 2023 Mar 6;14(3):179. doi: 10.1038/s41419-023-05687-2 (PMC9986231; doi:10.1038/s41419-023-05687-2)
Supplement: Supplementary file 1 — Supplemental file 1. Supplemental Figure S1-3. [file 41419_2023_5687_MOESM1_ESM.docx]

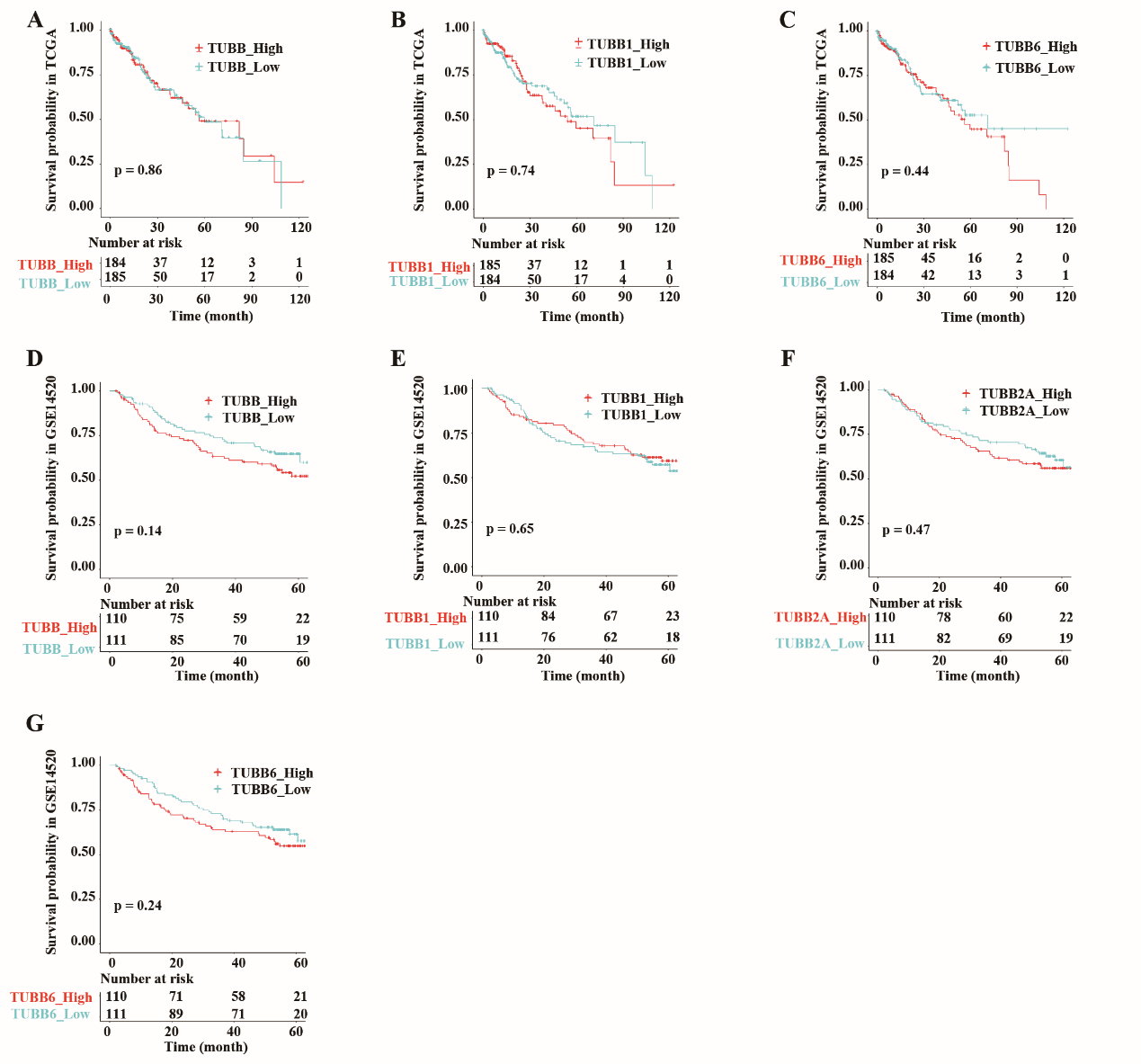


**Figure S1.** **Prognostic value of TUBBs in HCC patients from TCGA and GSE14520**. **A-C)** Kaplan-Meier (K-M) survival curves of TUBB, TUBB1, TUBB6 in HCC patients from TCGA. **D-G)** K-M survival curves of TUBB, TUBB1, TUBB2A, TUBB6 in HCC patients from

GSE14520.


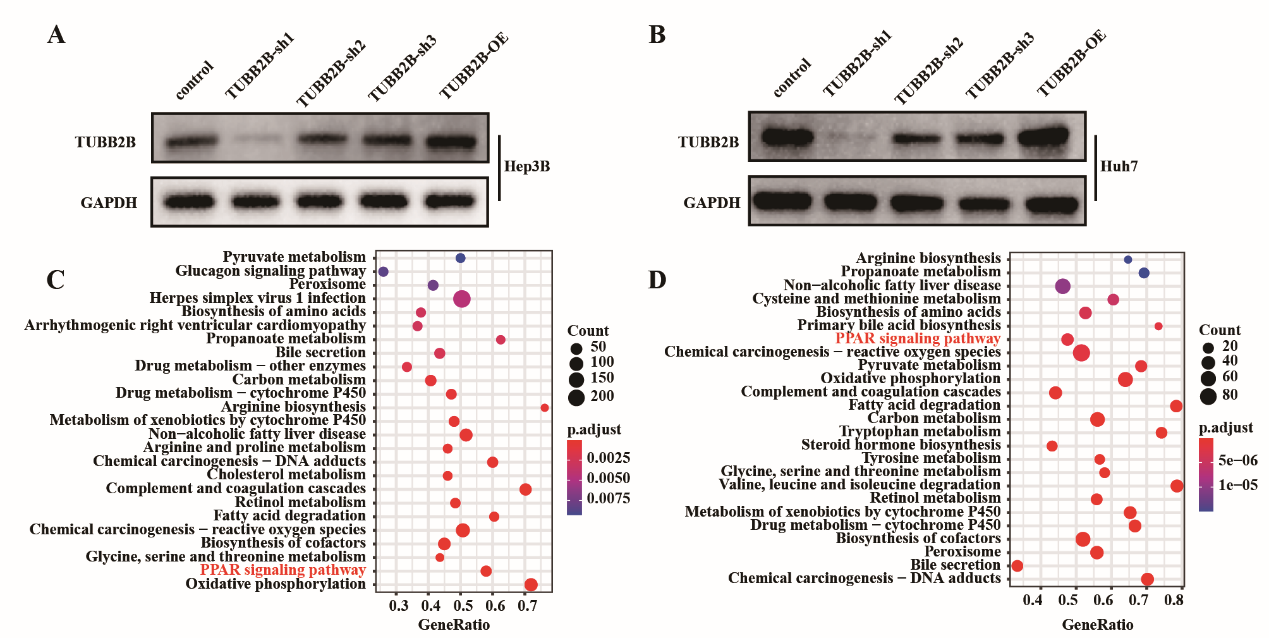


**Figure S2. A, B)** The interference and overexpression of TUBB2B were assessed by western blot. **C, D)** Bubble plot of the enriched KEGG pathways by GSEA stratified by TUBB2B in TCGA and GSE14520.


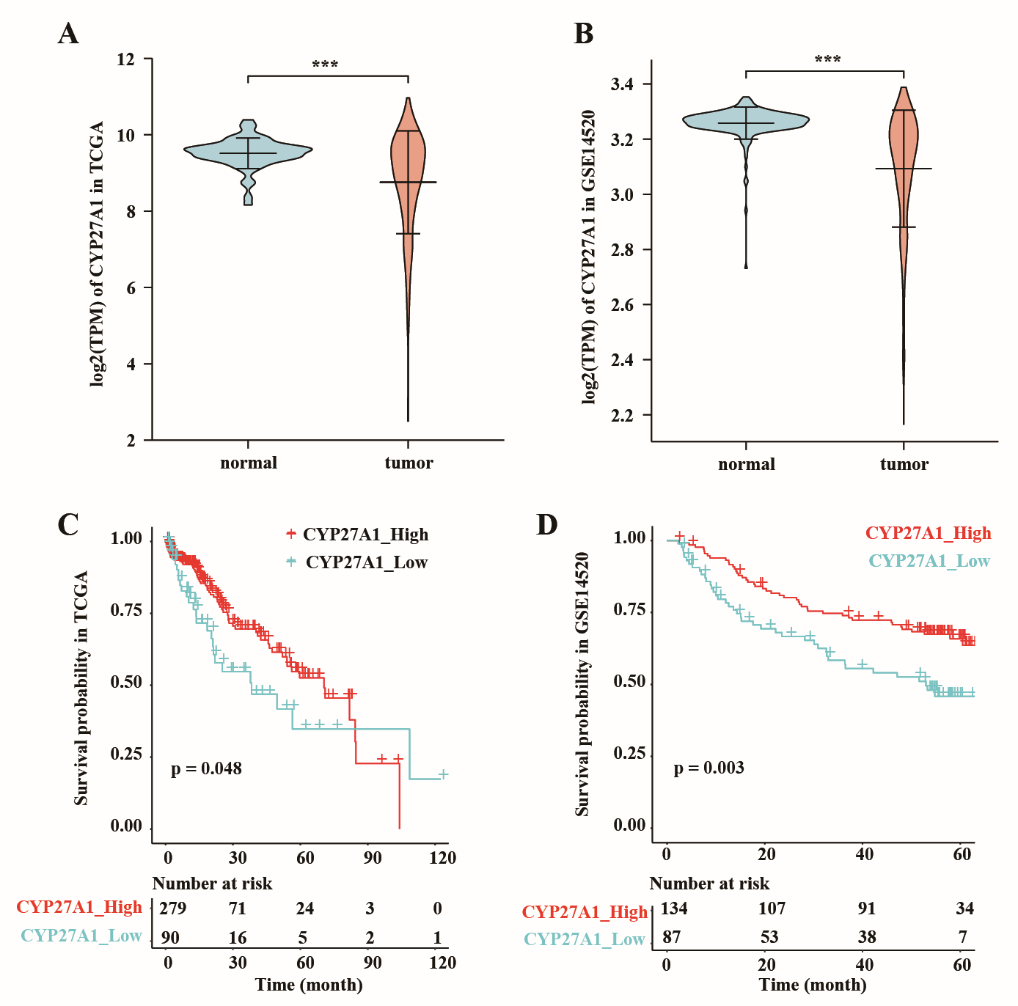


**Figure S3. Expression and prognostic value of CYP27A1 in HCC patients.** **A, B)** Violin plots showed the CYP27A1 expression in HCC from TCGA and GSE14520 data. **C, D)** The K-M survival curves of CYP27A1 in HCC from TCGA and GSE14520 data. ***p<0.001
