# Supplementary material for: TUBB2B facilitates progression of hepatocellular carcinoma by regulating cholesterol metabolism through targeting HNF4A/CYP27A1
Source: Cell Death Dis. 2023 Mar 6;14(3):179. doi: 10.1038/s41419-023-05687-2 (PMC9986231; doi:10.1038/s41419-023-05687-2)
Supplement: Supplementary file 2 — Supplemental file 2. Supplemental Table 1-7 [file 41419_2023_5687_MOESM2_ESM.docx]

**Supplementary Table 1 The clinical characteristics of 370 HCC patients in TCGA**

| Characteristic | Freq |
| --- | --- |
| gender, No. (%) | |
| Male | 249 (67%) |
| Female | 121 (33%) |
| Missing data | 0 (0%) |
| age, No. (%) | |
| > 60 | 193 (52%) |
| ≤ 60 | 177 (48%) |
| Body Mass Index, No. (%) | |
| > 26 | 131 (35%) |
| ≤ 26 | 203 (55%) |
| Missing data | 36 (10%) |
| Histological grade, No. (%) | |
| G1+G2 | 232 (63%) |
| G3+G4 | 133 (36%) |
| Missing data | 5 (1%) |
| Tumor stage, No. (%) | |
| Stage 1+2 | 256 (69%) |
| Stage 3+4 | 90 (24%) |
| Missing data | 24 (6%) |
| HBV, No. (%) | |
| N0 | 225 (61%) |
| Yes | 145 (39%) |
| Missing data | 0 (0%) |
| HCV, No. (%) | |
| N0 | 266 (72%) |
| Yes | 104 (28%) |
| Missing data | 0 (0%) |
| T, No. (%) |  |
| T1+T2 | 274 (74%) |
| T3+T4 | 93 (25%) |
| Missing data | 3 (1%) |
| N, No. (%) |  |
| N0 | 252 (68%) |
| N1+NX | 117 (32%) |
| Missing data | 1 (0%) |
| M, No. (%) | |
| M0 | 266 (72%) |
| M1+MX | 104 (28%) |
| Missing data | 0 (0%) |
| TUBB, No. (%) | |
| Low expression | 185 (50%) |
| High expression | 185 (50%) |
| TUBB1, No. (%) | |
| Low expression | 185 (50%) |
| High expression | 185 (50%) |
| TUBB2A, No. (%) | |
| Low expression | 185 (50%) |
| High expression | 185 (50%) |
| TUBB2B, No. (%) | |
| Low expression | 185 (50%) |
| High expression | 185 (50%) |
| TUBB3, No. (%) | |
| Low expression | 185 (50%) |
| High expression | 185 (50%) |
| TUBB6, No. (%) | |
| Low expression | 185 (50%) |
| High expression | 185 (50%) |

**Supplementary Table 2 The clinical characteristics of 221 HCC patients in GSE14520**

| Characteristic | Freq |
| --- | --- |
| gender, No. (%) | |
| Male | 191 (85%) |
| Female | 30 (13%) |
| Missing data | 4 (2%) |
| age, No. (%) | |
| > 60 | 40 (18%) |
| ≤ 60 | 181 (80%) |
| Missing data | 4 (2%) |
| HBV No. (%) | |
| AVR-CC | 56 (25%) |
| CC+NO | 162 (72%) |
| Missing data | 7 (3%) |
| ALT, No. (%) | |
| > 50U/L | 91 (40%) |
| ≤ 50U/L | 130 (58%) |
| Missing data | 4 (2%) |
| Tumor size, No. (%) | |
| > 5cm | 80 (36%) |
| ≤ 5cm | 140 (62%) |
| Missing data | 5 (2%) |
| Multinodular, No. (%) | |
| NO | 176 (78%) |
| Yes | 45 (20%) |
| Missing data | 4 (2%) |
| Cirrhosis, No. (%) | |
| NO | 18 (8%) |
| Yes | 203 (90%) |
| Missing data | 4 (2%) |
| TNM, No. (%) | |
| I+II | 170 (76%) |
| III+IV | 49 (22%) |
| Missing data | 6 (3%) |
| BCLC stage, No. (%) | |
| 0+A | 168 (75%) |
| B+C | 51 (23%) |
| Missing data | 6 (3%) |
| alpha-fetoprotein, No. (%) | |
| > 300U/L | 100 (44%) |
| ≤ 300U/L | 118 (52%) |
| Missing data | 7 (3%) |
| TUBB, No. (%) | |
| Low expression | 113 (50%) |
| High expression | 112 (50%) |
| TUBB1, No. (%) | |
| Low expression | 113 (50%) |
| High expression | 112 (50%) |
| TUBB2A, No. (%) | |
| Low expression | 113 (50%) |
| High expression | 112 (50%) |
| TUBB2B, No. (%) | |
| Low expression | 113 (50%) |
| High expression | 112 (50%) |
| TUBB3, No. (%) | |
| Low expression | 113 (50%) |
| High expression | 112 (50%) |
| TUBB6, No. (%) | |
| Low expression | 113 (50%) |
| High expression | 112 (50%) |

**Supplementary Table 3 Univariate and multivariate analysis of overall survival in 311 HCC patients of TCGA**

| characteristics | univariate analysis | | | multivariate analysis | | |
| --- | --- | --- | --- | --- | --- | --- |
|  | Hazard  Ratio | CI95 | P.Value | Hazard  Ratio | CI95 | P.Value |
| age | 1.03 | 1.01-1.04 | 0.004* | 1.02 | 1-1.04 | 0.017* |
| BMI | 1.03 | 1-1.06 | 0.093 | 1.01 | 0.97-1.04 | 0.662 |
| gender | 1.32 | 0.86-2.02 | 0.199 |  |  |  |
| grade | 1.13 | 0.91-1.41 | 0.259 |  |  |  |
| HBV | 1.4 | 0.91-2.15 | 0.122 |  |  |  |
| HCV | 1.74 | 1.07-2.83 | 0.026* | 1.48 | 0.8-2.75 | 0.213 |
| T | 1.35 | 1.07-1.69 | 0.01* | 1.34 | 1.04-1.75 | 0.026* |
| M | 2.4 | 1.56-3.69 | <0.001* | 2.06 | 1.05-4.03 | 0.036* |
| N | 1.83 | 1.18-2.84 | 0.007* | 0.82 | 0.43-1.56 | 0.549 |
| AJCC stage | 1.22 | 0.96-1.57 | 0.11 |  |  |  |
| TUBB | 1 | 1-1 | 0.986 |  |  |  |
| TUBB1 | 1.48 | 0.56-3.89 | 0.429 |  |  |  |
| TUBB2A | 1 | 1-1 | 0.196 |  |  |  |
| TUBB2B | 1.06 | 1.02-1.1 | 0.004* | 1.05 | 1.01-1.1 | 0.022* |
| TUBB3 | 1.05 | 0.9-1.23 | 0.546 |  |  |  |
| TUBB6 | 1 | 0.99-1.01 | 0.965 |  |  |  |

HCC: hepatocellular carcinoma; CI: confidence interval. * p<0.05

**Supplementary Table 4 Univariate and multivariate analysis of overall survival in 221 HCC patients of GSE14520**

| characteristics | univariate analysis | | | multivariate analysis | | |
| --- | --- | --- | --- | --- | --- | --- |
|  | Hazard  Ratio | CI95 | P.Value | Hazard  Ratio | CI95 | P.Value |
| AFP | 1.63 | 1.06-2.5 | 0.025* | 1.24 | 0.78-1.97 | 0.364 |
| age | 0.99 | 0.97-1.01 | 0.405 |  |  |  |
| ALT | 1.08 | 0.7-1.66 | 0.727 |  |  |  |
| BCLC stage | 3.55 | 2.27-5.54 | <0.001* | 3.59 | 1.59-8.14 | 0.002* |
| Cirrhosis | 4.62 | 1.14-18.8 | 0.032* | 4.38 | 1.06-18.14 | 0.041* |
| gender | 0.59 | 0.28-1.22 | 0.153 |  |  |  |
| HBV | 0.75 | 0.47-1.19 | 0.219 |  |  |  |
| Multinodular | 1.59 | 0.99-2.57 | 0.057 | 0.4 | 0.2-0.77 | 0.006* |
| TNM | 3.52 | 2.24-5.51 | <0.001* | 1.89 | 0.89-4.03 | 0.098 |
| Tumor_Size | 1.92 | 1.25-2.96 | 0.003* | 1.02 | 0.56-1.86 | 0.95 |
| TUBB | 1.54 | 1.11-2.14 | 0.011* | 1.74 | 1.15-2.62 | 0.009* |
| TUBB1 | 1.49 | 0.64-3.45 | 0.357 |  |  |  |
| TUBB2A | 1.1 | 0.9-1.34 | 0.336 |  |  |  |
| TUBB2B | 1.36 | 1.1-1.69 | 0.005* | 1.33 | 1.03-1.71 | 0.029* |
| TUBB3 | 1.38 | 0.95-2 | 0.091 | 0.69 | 0.42-1.13 | 0.141 |
| TUBB6 | 1.18 | 0.95-1.45 | 0.131 |  |  |  |

HCC: hepatocellular carcinoma; CI: confidence interval. * p<0.05

**Supplementary Table 5 Primer sequences**

| **Gene** | **primer sequences** |
| --- | --- |
| TUBB2B forward | AGGCACCATGCGTGAGAT |
| TUBB2B reverse | TCCAGCTGCAAA TCACTGTC |
| CYP27A1 forward | ACTCCCGGATCATAGAAAAGG |
| CYP27A1 reverse | TGGAAGCTTTCAGGCTCAG |
| HMGCS2 forward | AAGTCTCTGGCTCGCCTGATGT |
| HMGCS2 reverse | TCCAGGTCCTTGTTGGTGTAGG |
| PCK2 forward | AGCCTCTTCCACCTGGTGTT |
| PCK2 reverse | AATCGAGAGTTGGGATGTGC |
| SLC27A2 forward | GTATTGTGGCTGGTGCTACTC |
| SLC27A2 reverse | CCGAAGCAGTTCACCGATA |
| APOC3 forward | GGGATCCCCACCATGCAGCCCCGG |
| APOC3 reverse | GGAATTCTCAGGCAGCCACGGCTG |
| β-ACTIN forward | GATCATTGCTCCTCCTGAGC |
| β-ACTIN reverse | ACTCCTGCTTGCTGATCCAC |
| GAPDH forward | GGAGCGAGATCCCTCCAAAAT |
| GAPDH reverse | GGCTGTTGTCATACTTCTCATGG |

**Supplementary Table 6 Targeting sequences of shRNA and overexpression**

| **Gene** | **Targeting sequences** |
| --- | --- |
| sh-TUBB2B #1 | GCTGGAGAGAATCAATGTT |
| sh-TUBB2B #2 | GGCACGATGGATTCGGTTA |
| sh-TUBB2B #3 | CACTGGCAGTTACCATGGA |
| sh-HNF4A | GCAGGAACATATGGGAACCAA |
| sh-CYP27A1 | GCTTTCAATGAGGTGATTGAT |
| hTUBB2B-F(piggybac) | CGCCGGCGCCTTGGATGTGCACGATCTCACGCAT |
| hTUBB2B-R(piggybac) | CGCCGGCGCCTTGGATGTGCACGATCTCACGCAT |
| hHNF4a-F(piggybac) | GCGGCCGCATGCGACTCTCCAAAACCCTCGTCG |
| hHNF4a-R(piggybac) | TGCGCAGCTGCTCCCAAAACCTCTCAGCGTA |
| hCYP27A1-F(piggybac) | CTCGAGATGGCTGCGCTGGGCTGCGCGAGGC |
| hCYP27A1-R(piggybac) | CGCCGGCGCGGAGCGCGTCGGGTCGCGTCGGTA |

**Supplementary Table 7 Antibodies information**

| Gene | Details |
| --- | --- |
| TUBB2B | Abcam, Cat#ab179512, UK |
| CYP27A1 | Abcam, Cat#ab126785, UK |
| HNF4A | Abcam, Cat#ab200654, UK |
| BCL2 | Abcam, Cat#ab182858, UK |
| BAX | Abcam, Cat#ab182733, UK |
| Caspase-3 | Abcam, Cat#ab184787, UK |
| GAPDH | Abcam, Cat# ab8245, UK |
| β-actin | Abcam, Cat#ab8226, UK |
| secondary anti-rabbit IgG | Abcam, Cat#ab172730, UK |
| secondary anti-mouse IgG | Abcam, Cat#ab133470, UK |
