# Supplementary material for: TUBB2B facilitates progression of hepatocellular carcinoma by regulating cholesterol metabolism through targeting HNF4A/CYP27A1
Source: Cell Death Dis. 2023 Mar 6;14(3):179. doi: 10.1038/s41419-023-05687-2 (PMC9986231; doi:10.1038/s41419-023-05687-2)
Supplement: Supplementary file 3 — Supplemental file 3. Full and uncropped Western blots [file 41419_2023_5687_MOESM3_ESM.docx]

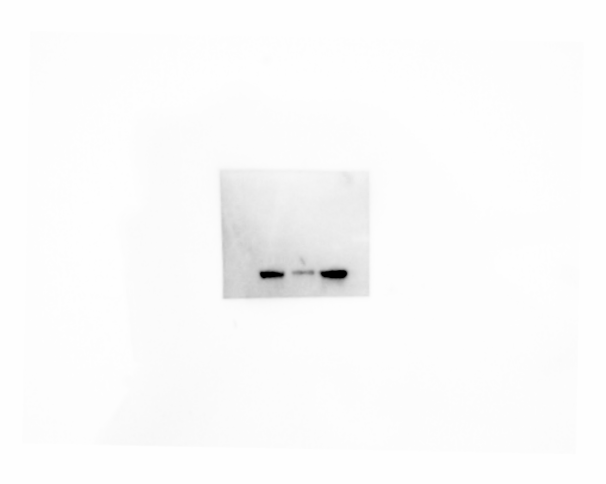

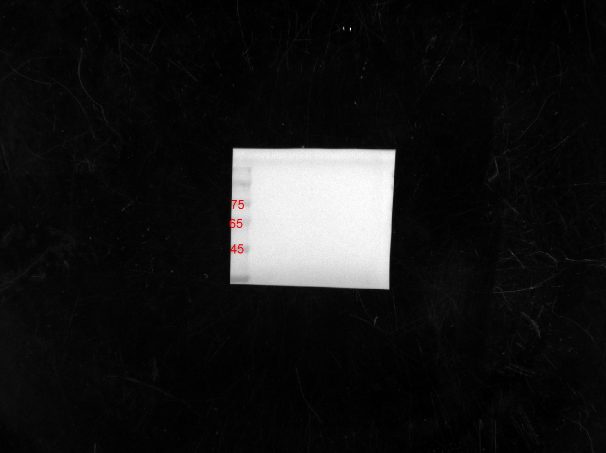


Figure 2E TUBB2B


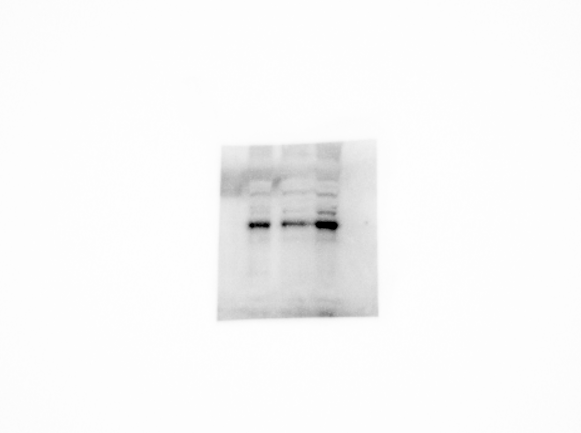

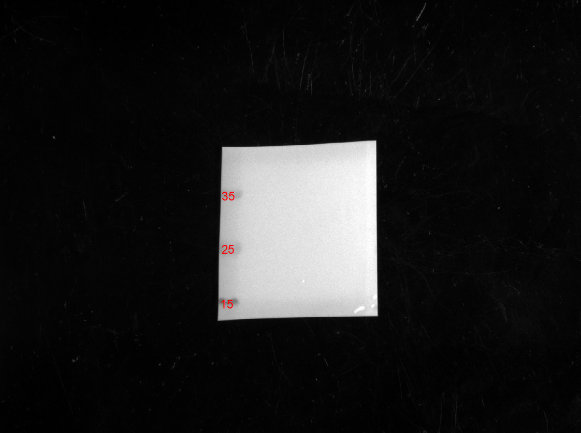


Figure 2E BCL2


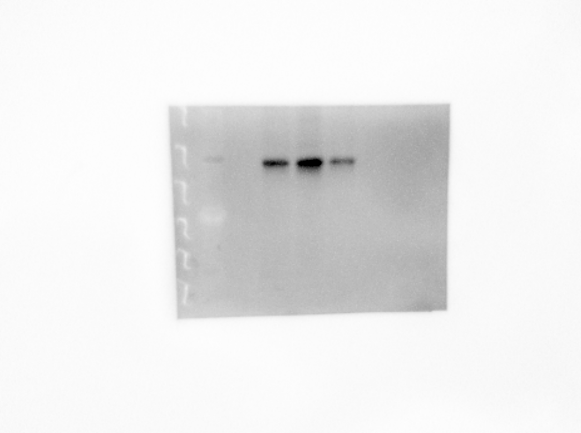

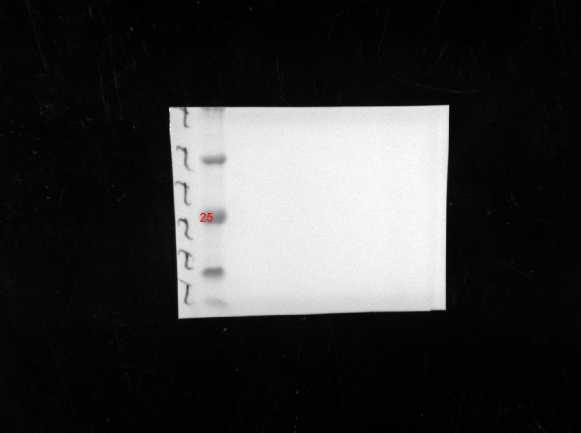


Figure 2E Caspase 3


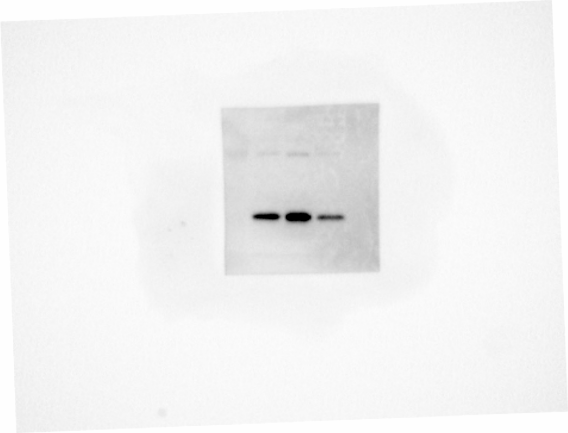

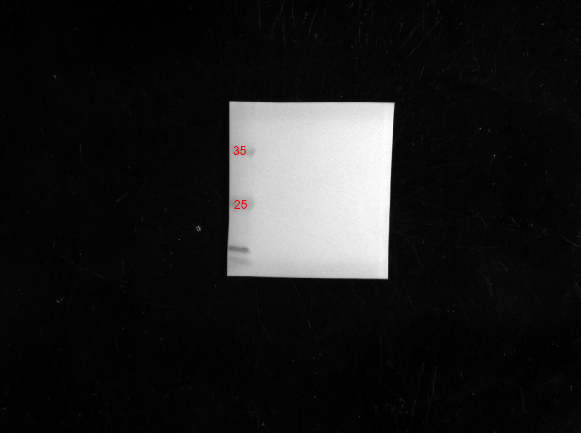


Figure 2E BAX


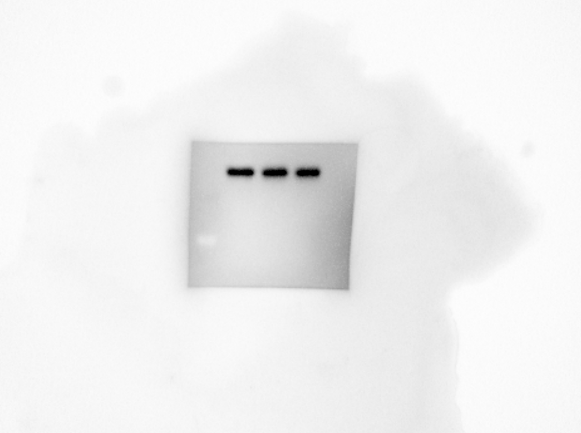

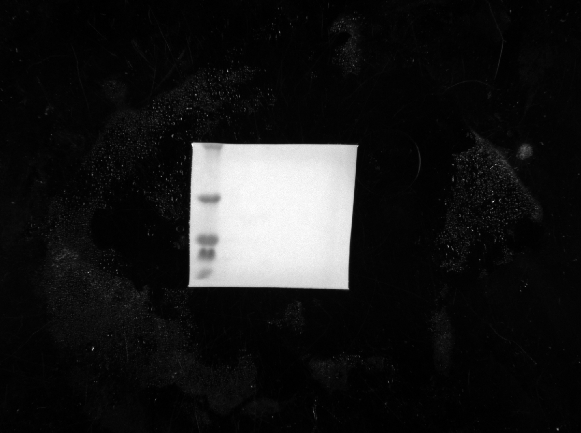


Figure 2E GAPDH


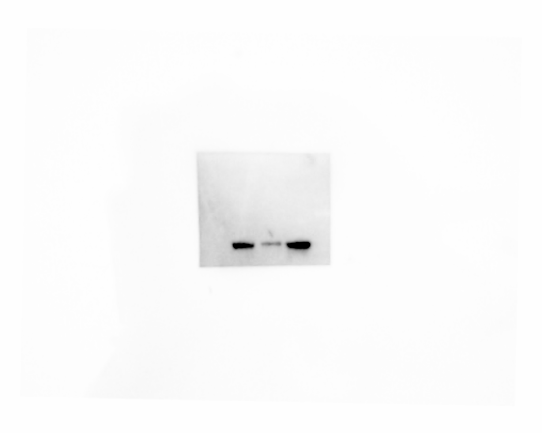

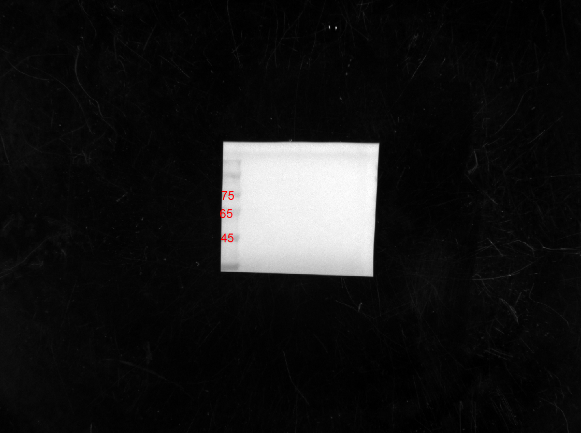


Figure 2F TUBB2B


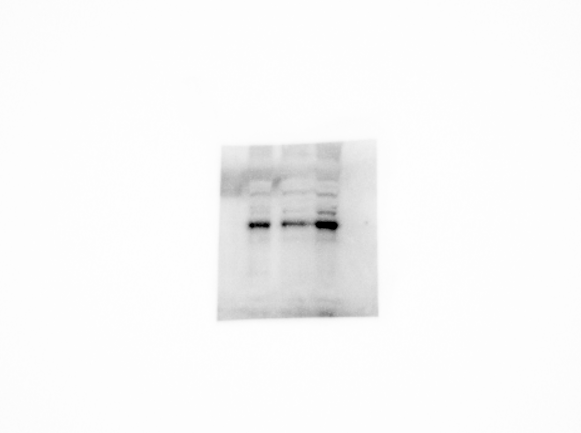

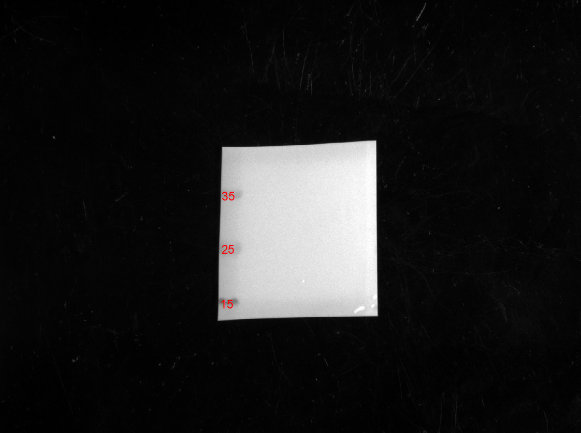


Figure 2F BCL2


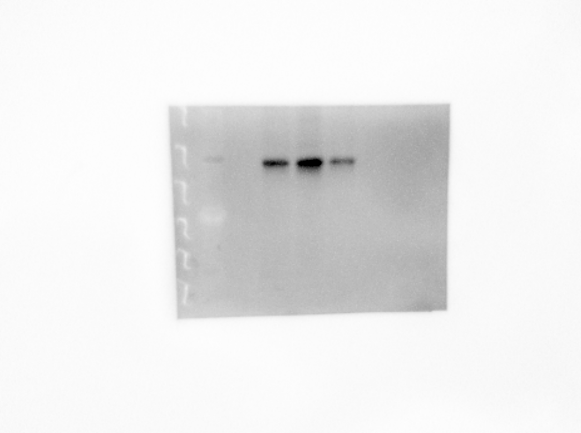

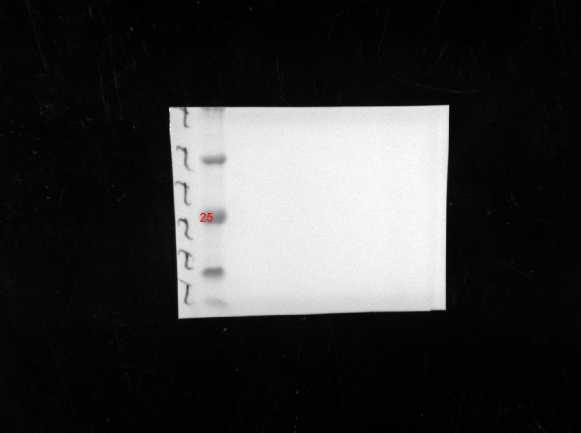


Figure 2F Caspase 3


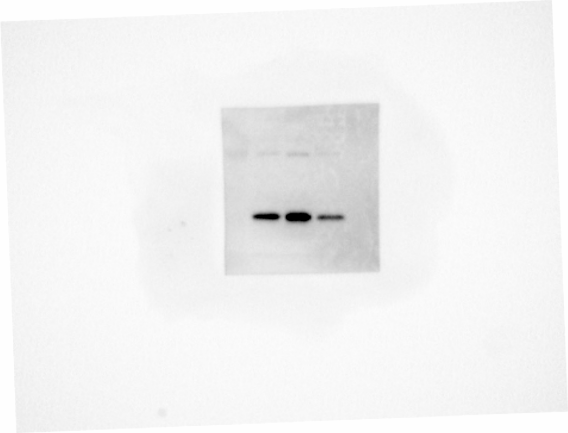

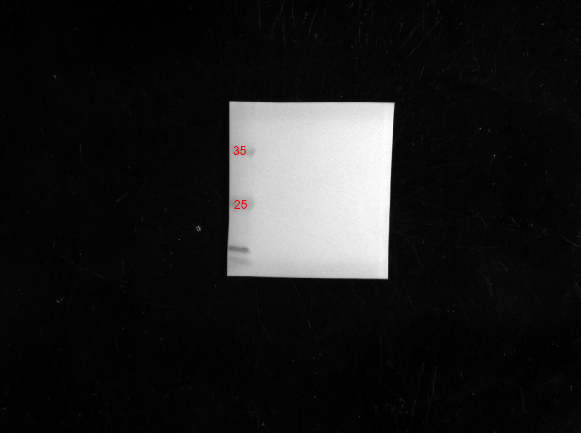


Figure 2F BAX


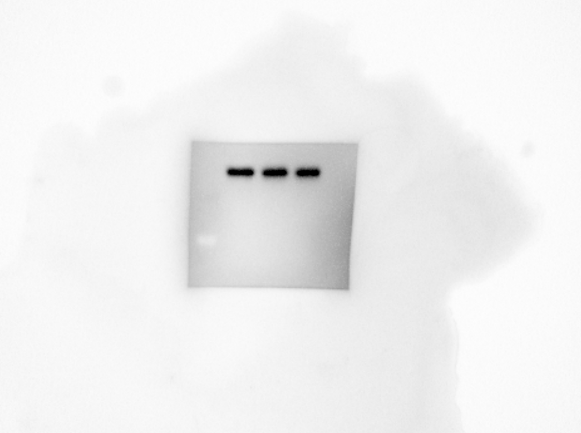

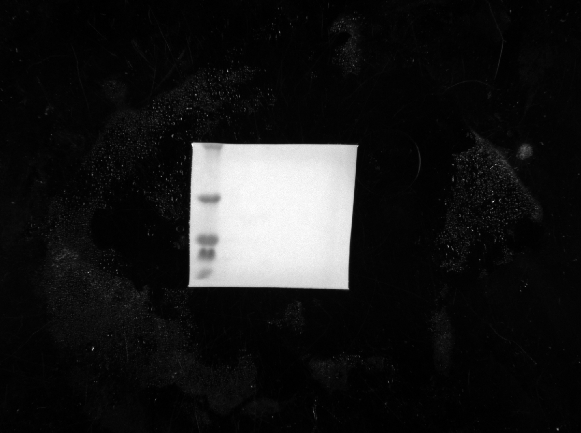


Figure 2F GAPDH


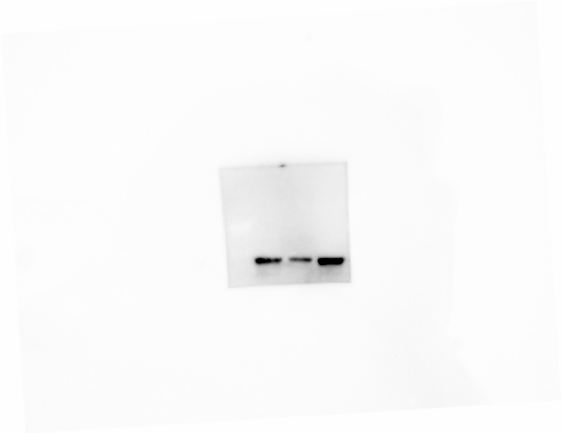

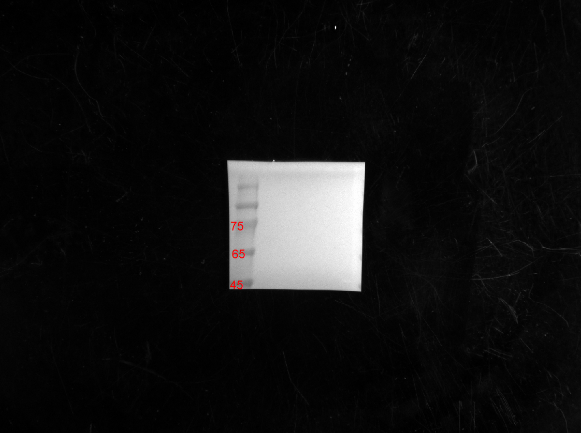


Figure 3E TUBB2B


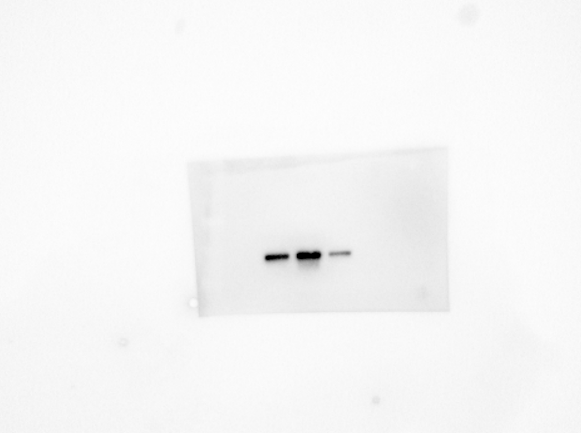

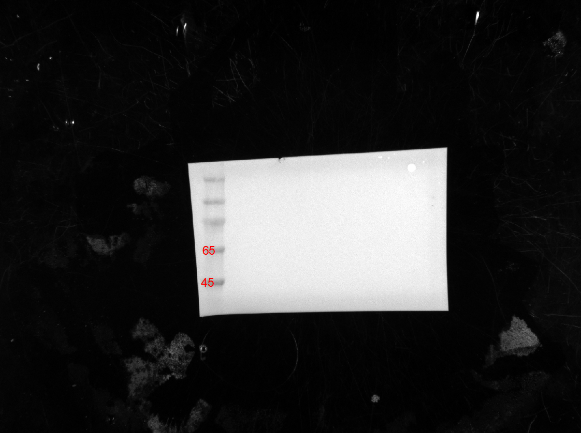


Figure 3E CYP27A1


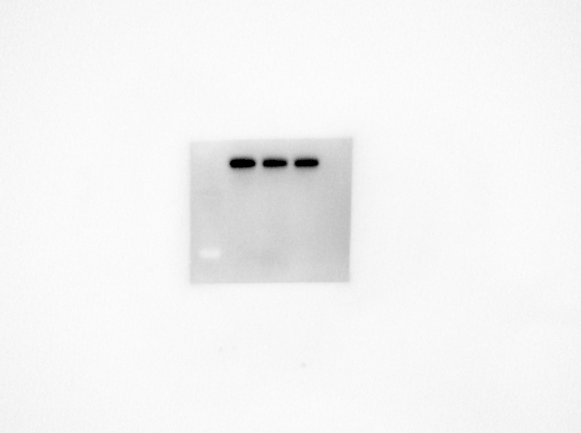

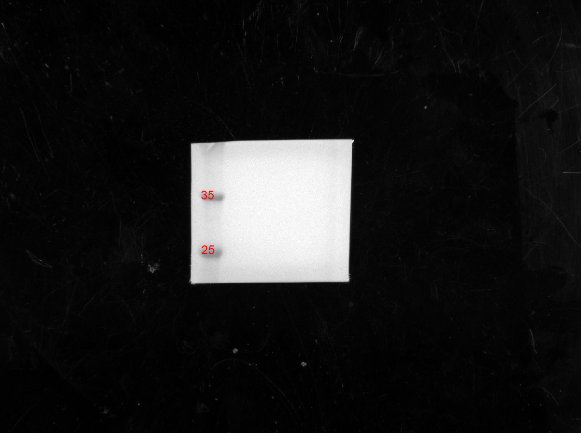


Figure 3E GAPDH


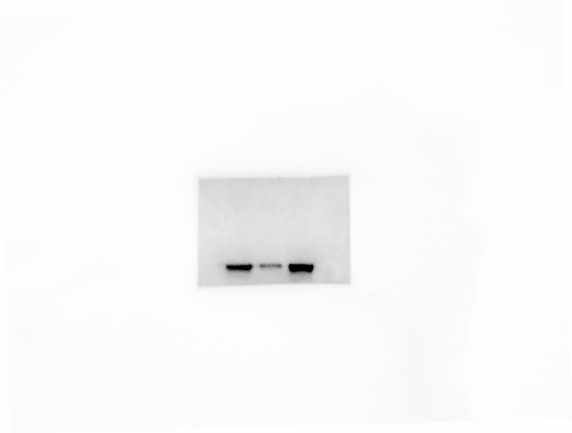

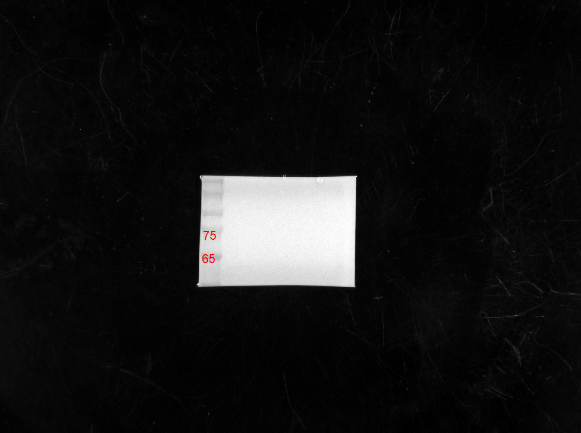


Figure 3F TUBB2B


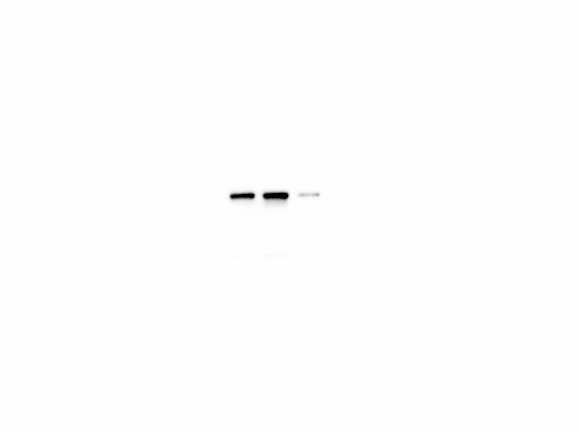

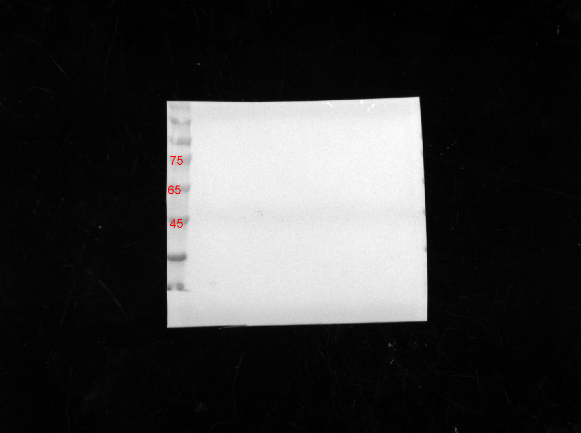


Figure 3F CYP27A1


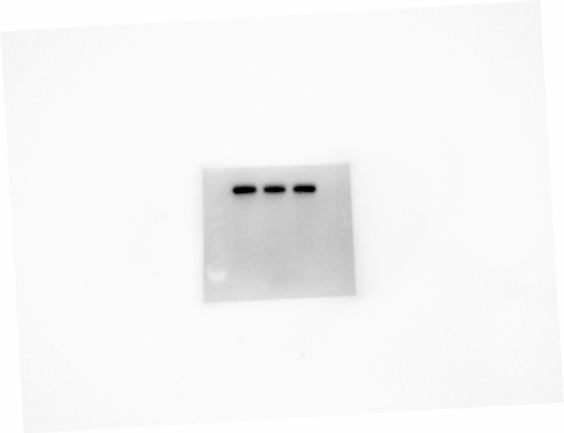

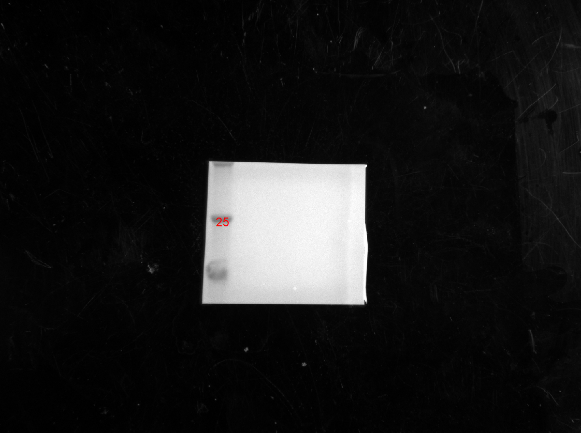


Figure 3F GAPDH


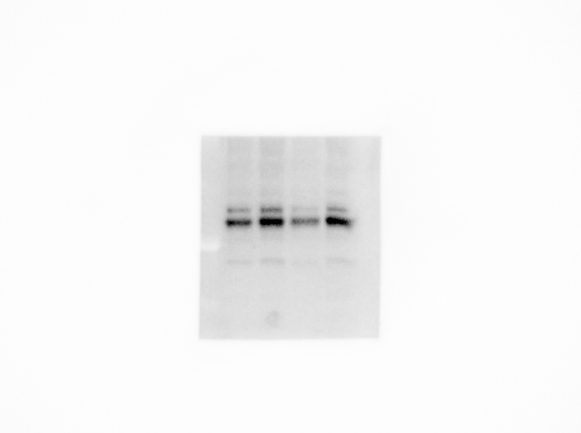

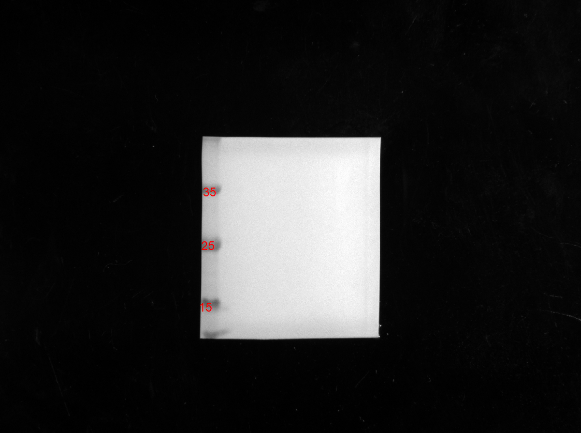


Figure 4G BCL2


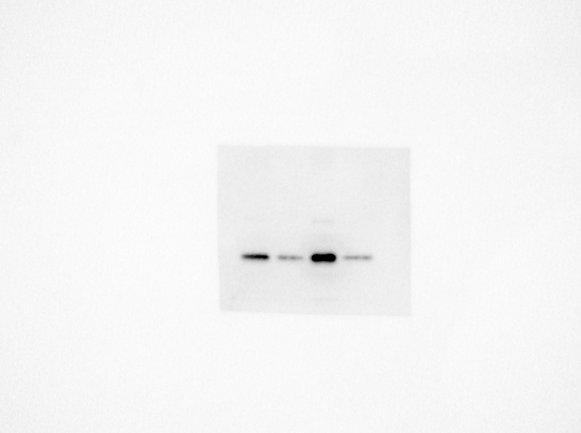

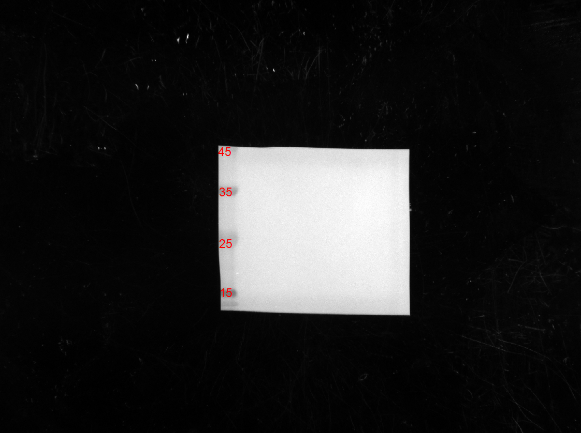


Figure 4G BAX


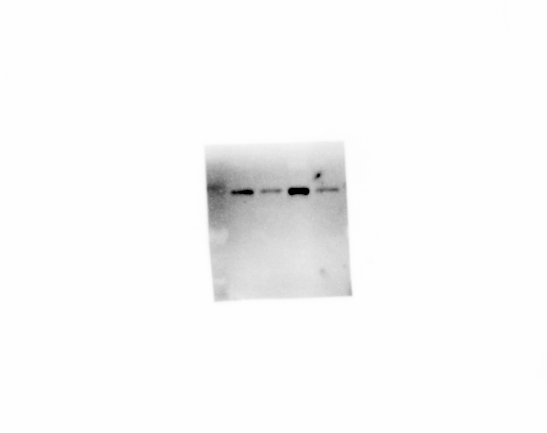

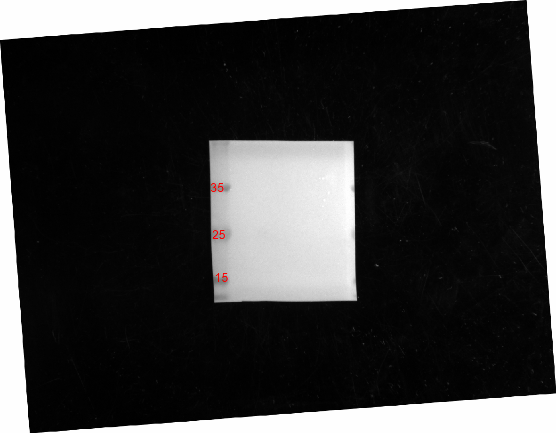


Figure 4G Caspase 3


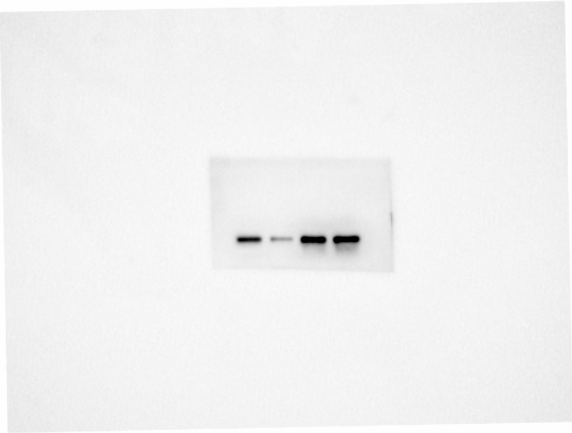

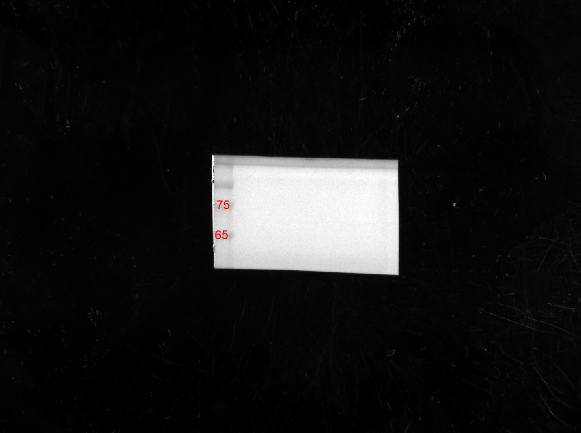


Figure 4G CYP27A1


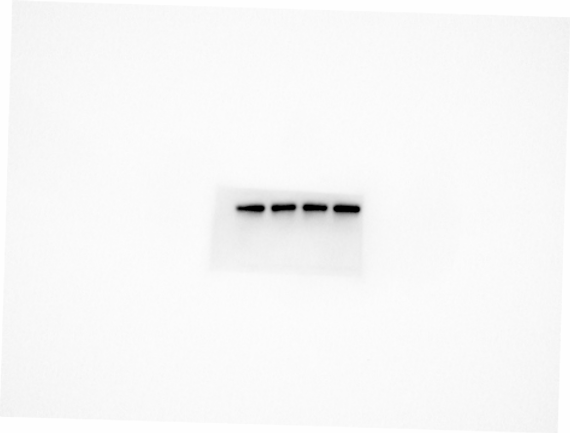

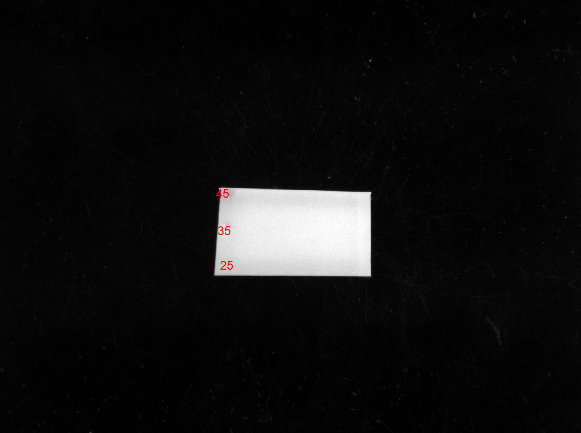


Figure 4G GAPDH


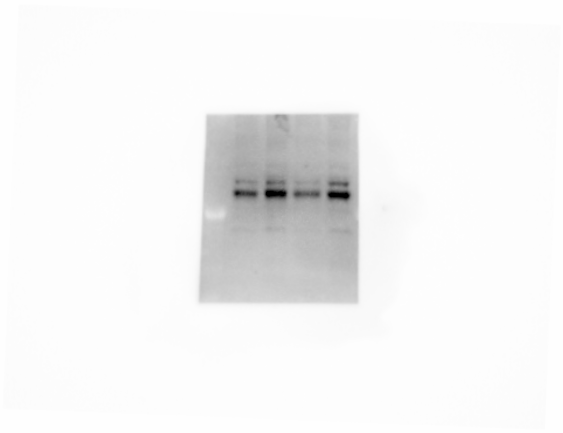

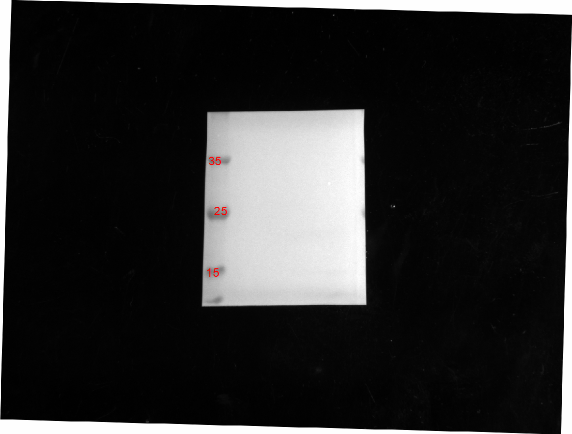


Figure 4H BCL2


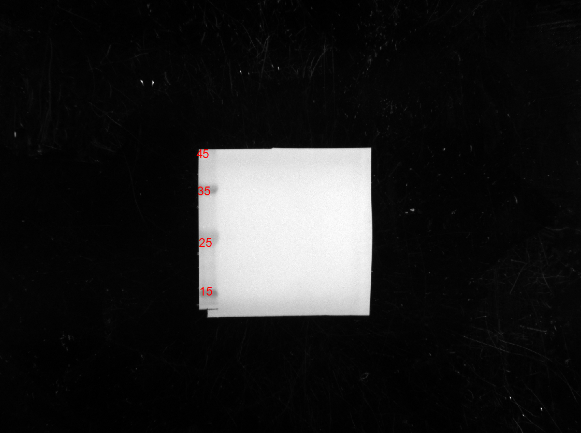

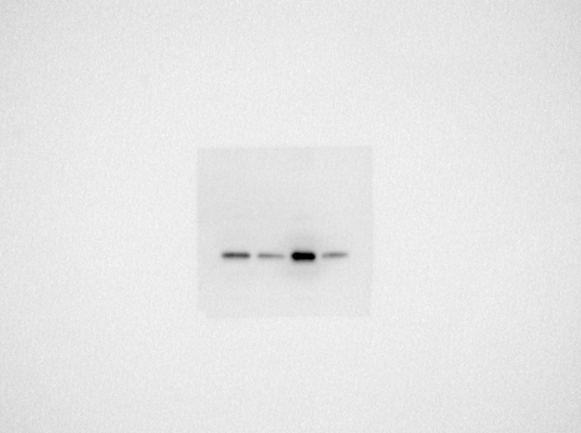


Figure 4H BAX


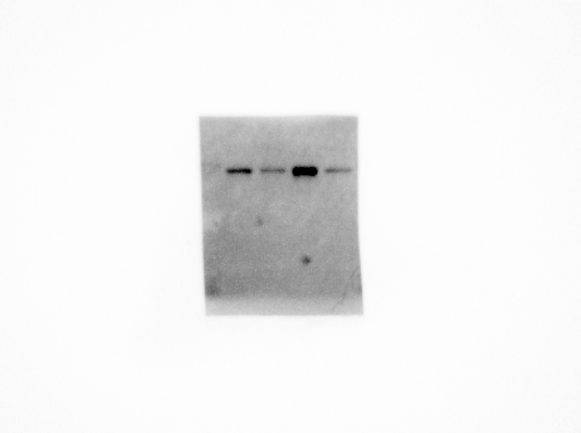

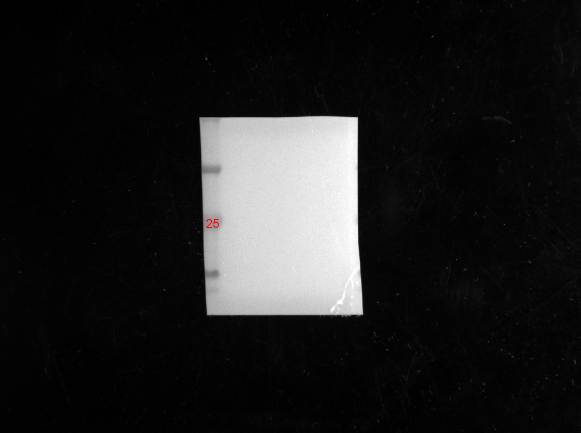


Figure 4H Caspase 3


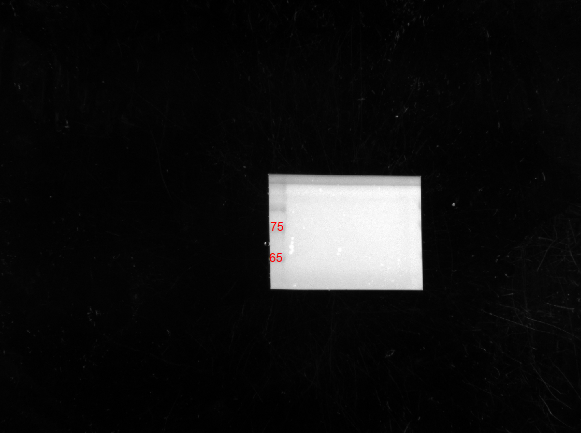

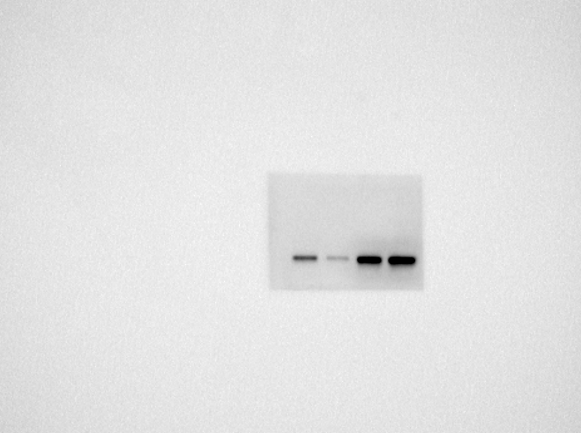


Figure 4H CYP27A1


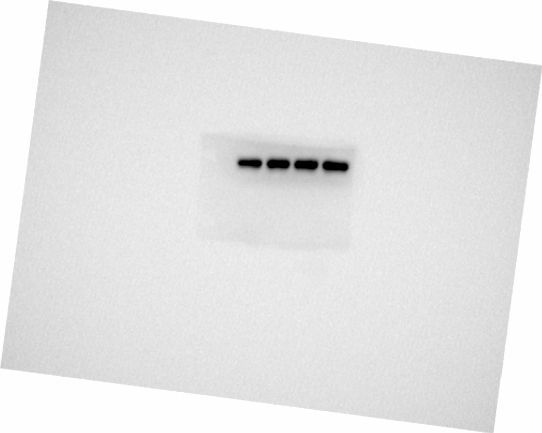

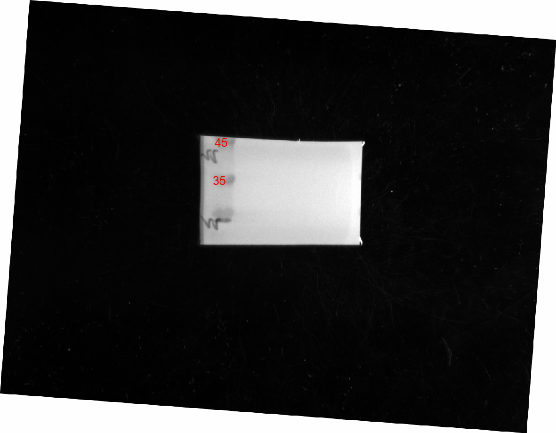


Figure 4H GAPDH


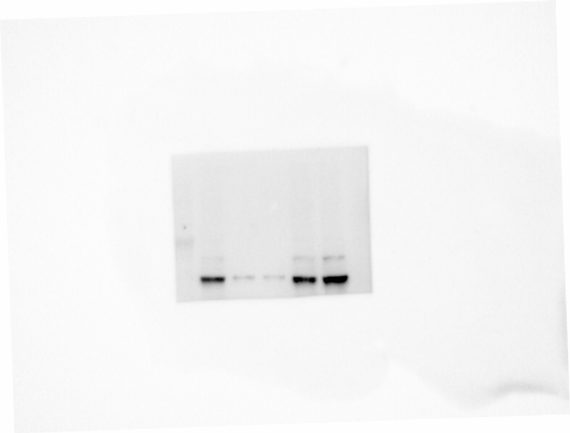

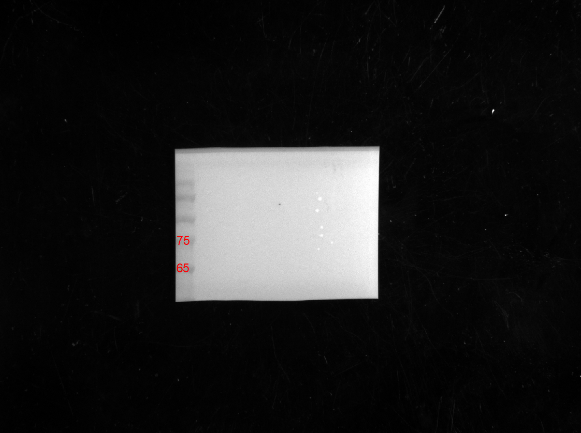


Figure 5G TUBB2B-Hep 3B


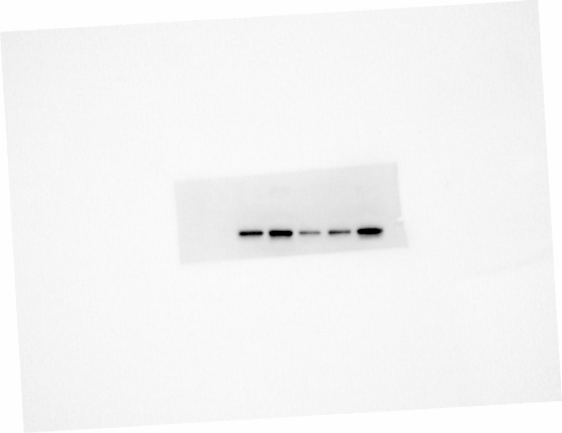

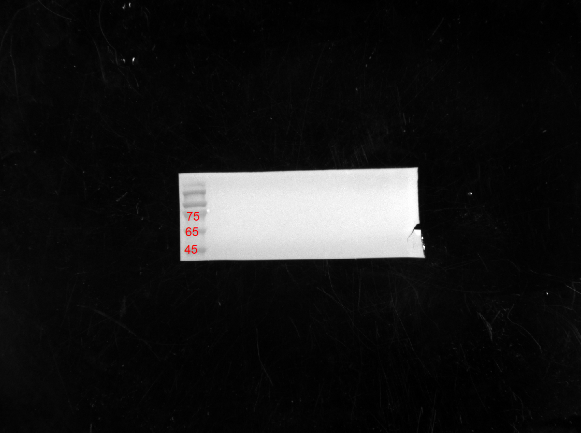


Figure 5G CYP27A1-Hep 3B


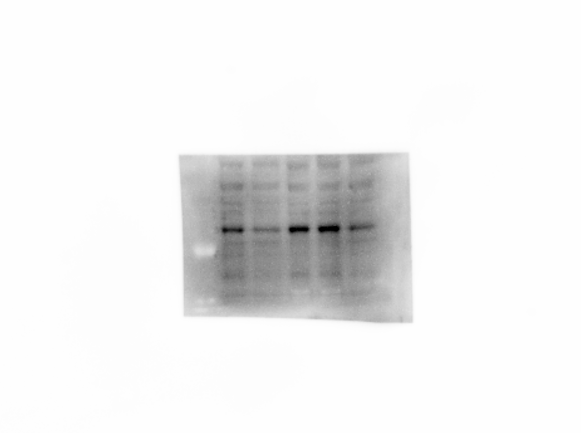

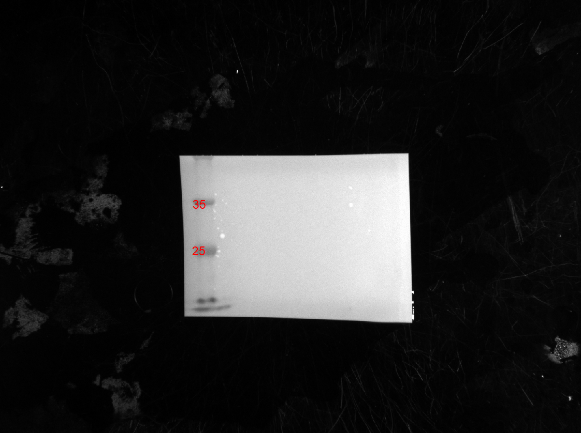


Figure 5G BCL2-Hep 3B


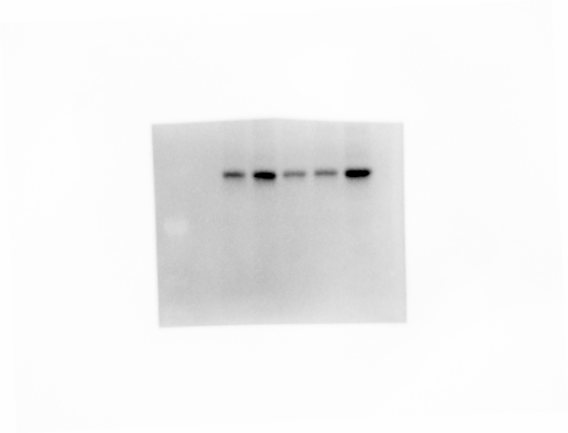

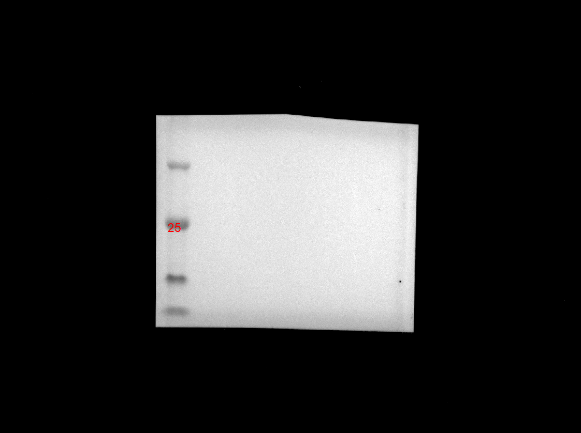


Figure 5G Caspase3-Hep 3B


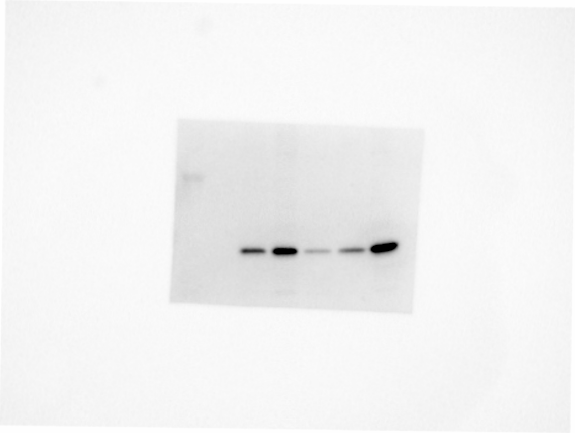

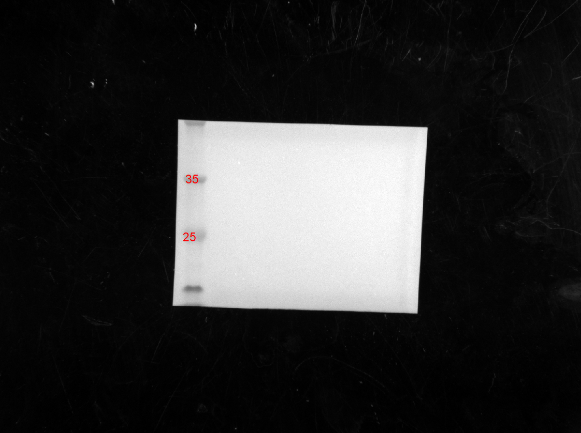


Figure 5G BAX -Hep 3B


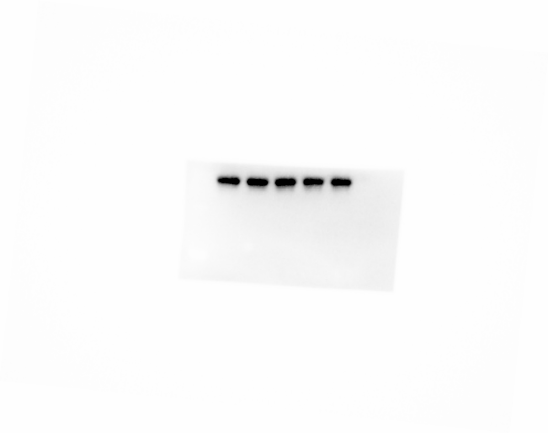

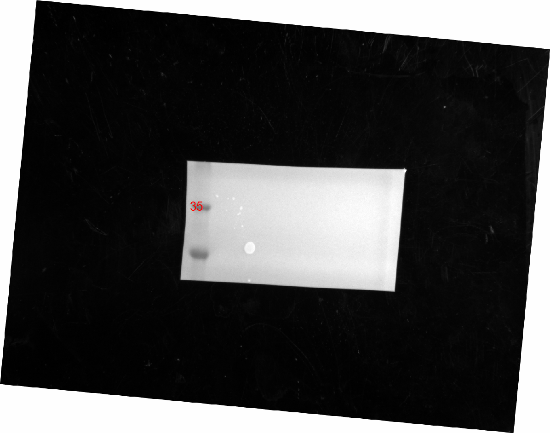


Figure 5G GAPDH-Hep 3B


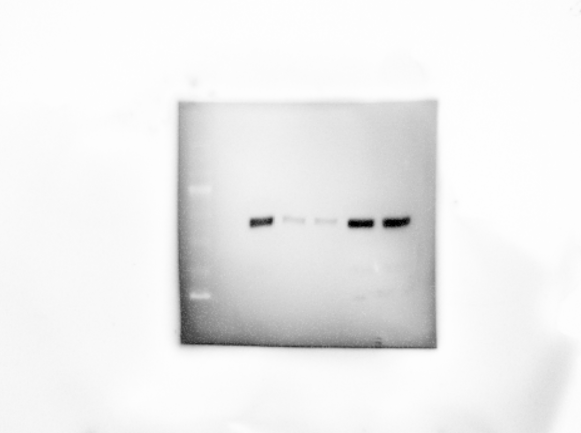

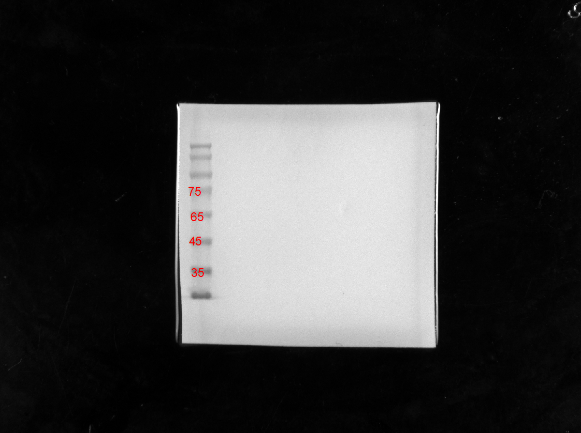


Figure 5G TUBB2B-Huh 7


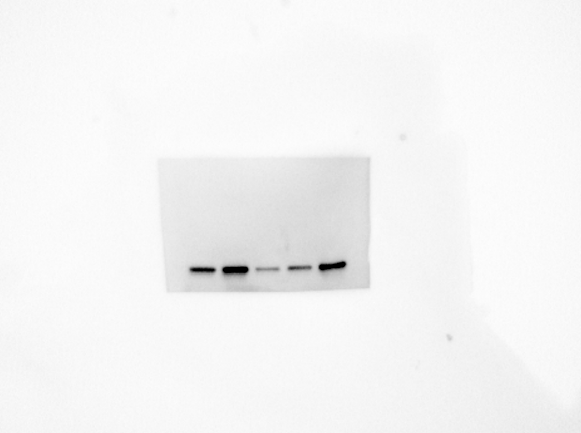

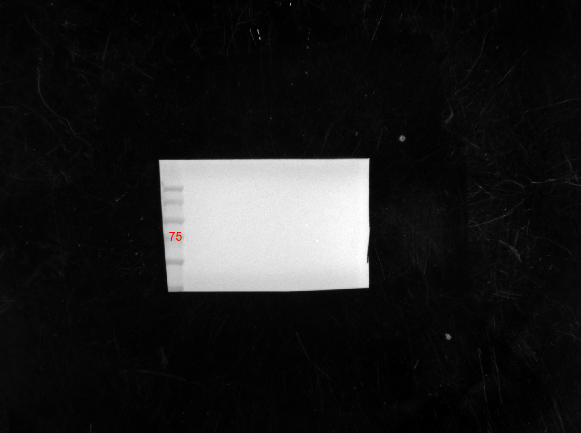


Figure 5G CYP27A1-Huh 7


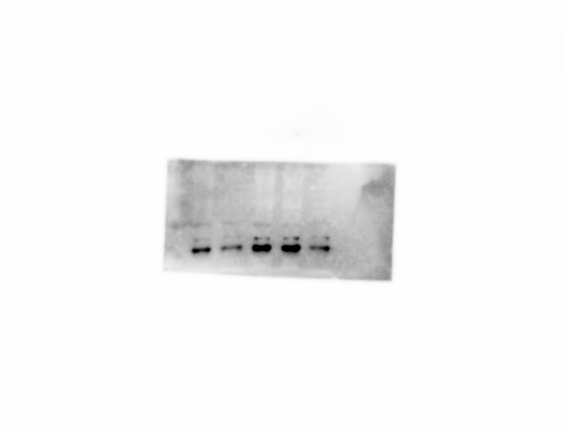

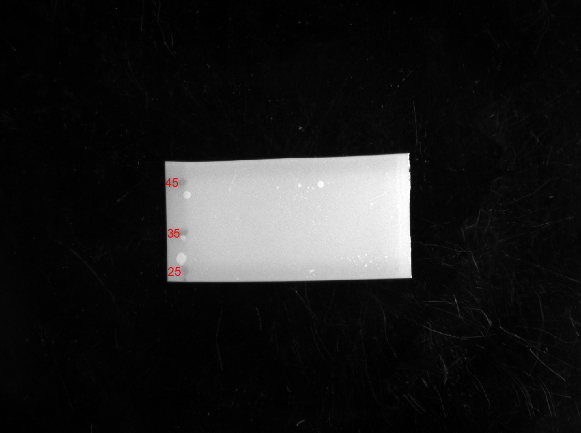


Figure 5G BCL2-Huh 7


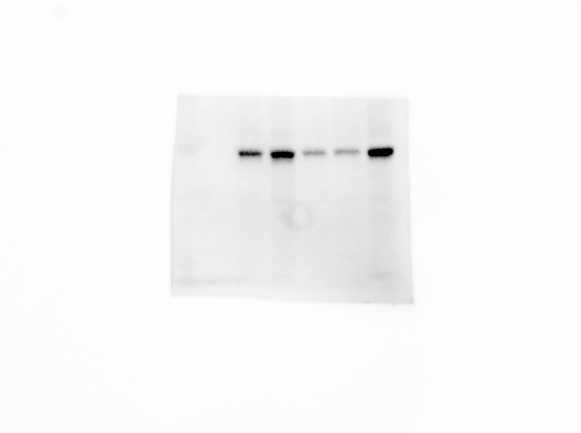

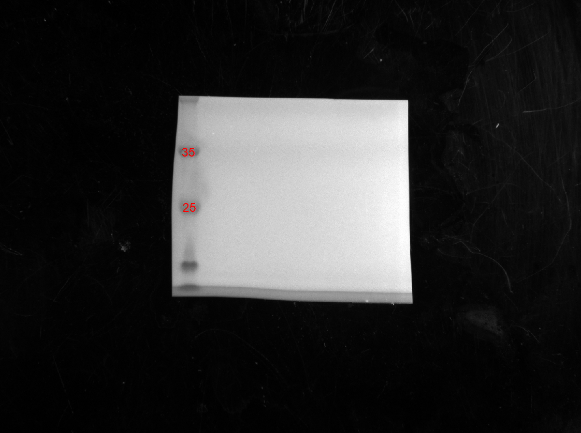


Figure 5G Caspase3- Huh 7


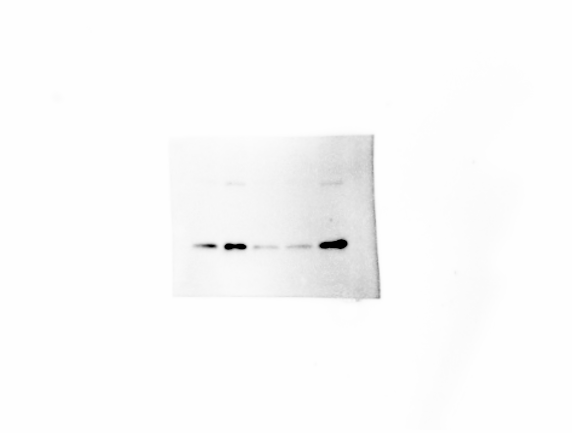

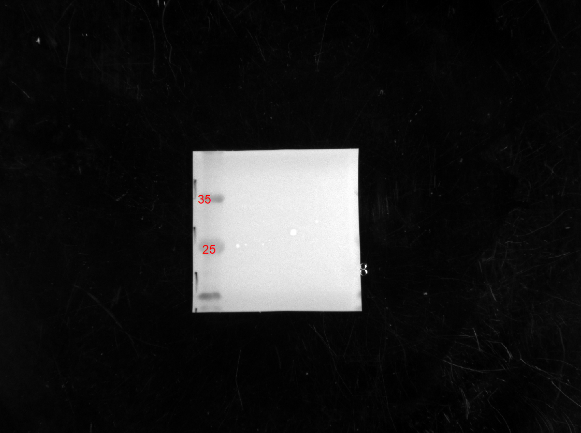


Figure 5G BAX -Huh 7


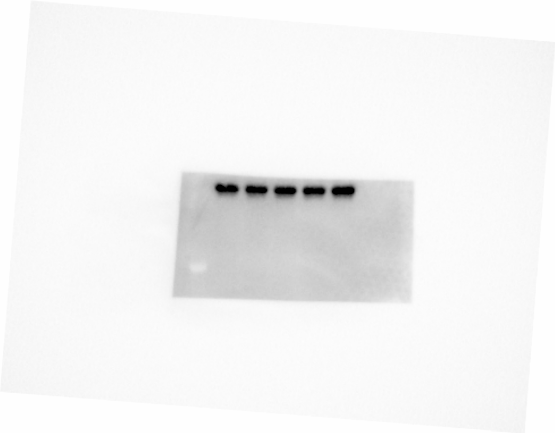

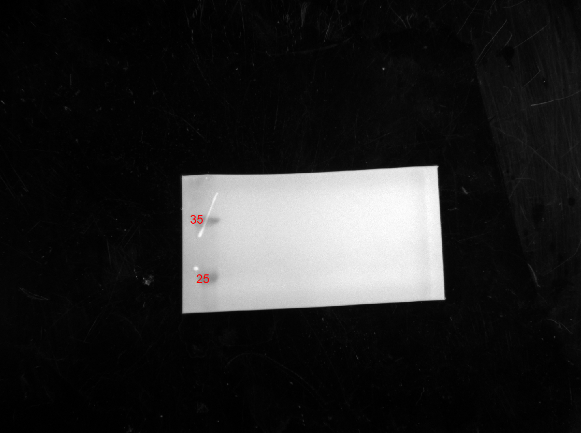


Figure 5G GAPDH-Huh 7


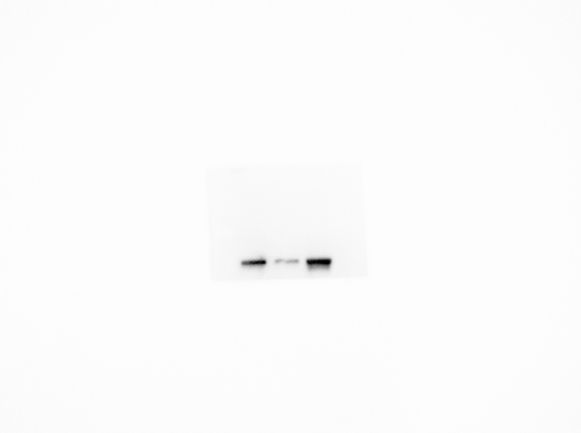

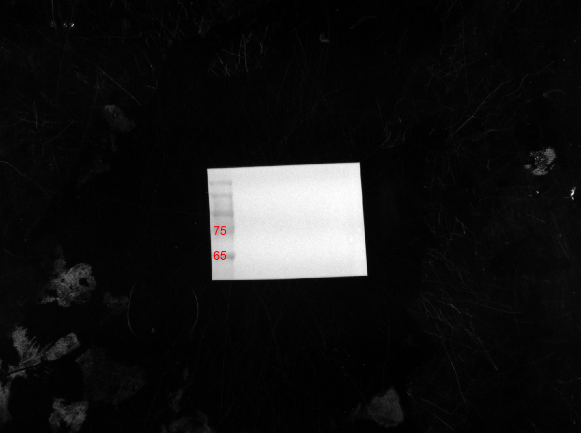


Figure 6C HNF4A


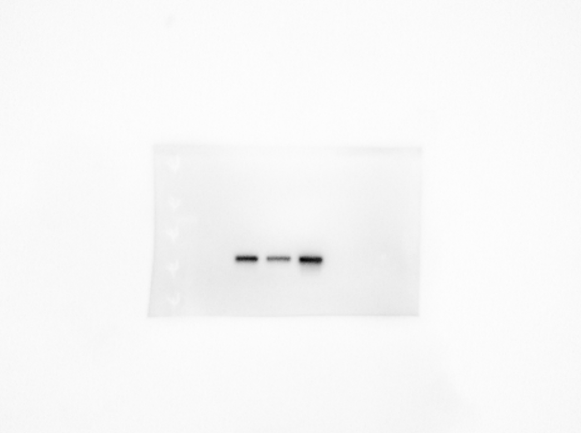

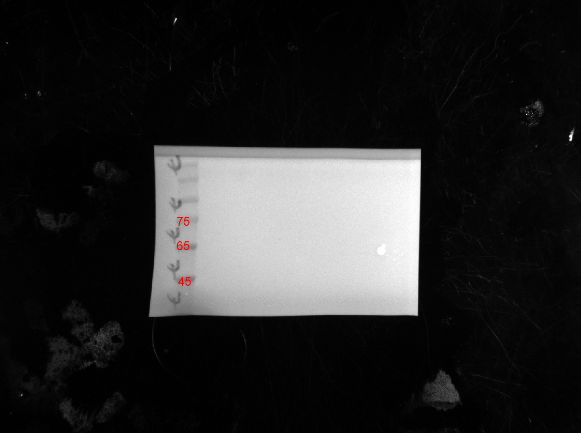


Figure 6C CYP27A1


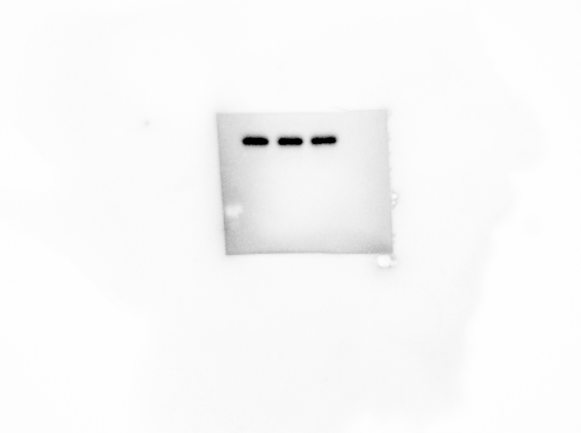

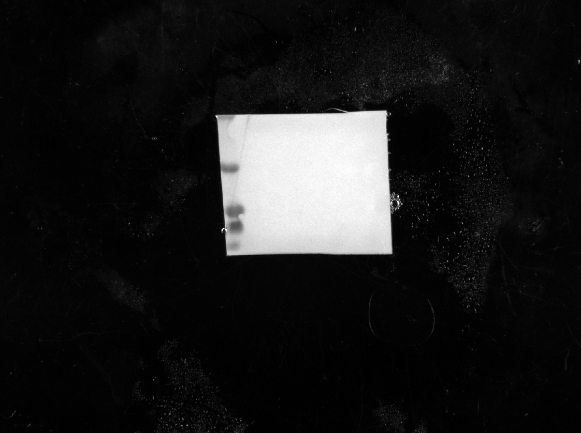


Figure 6C GAPDH


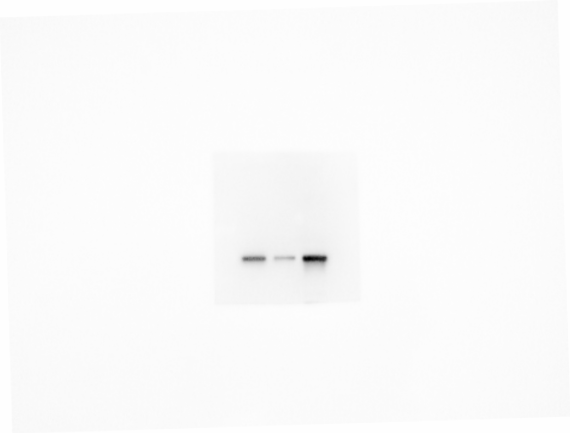

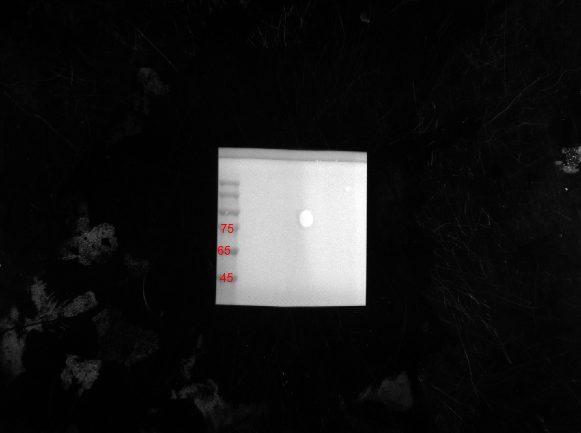


Figure 6D HNF4A


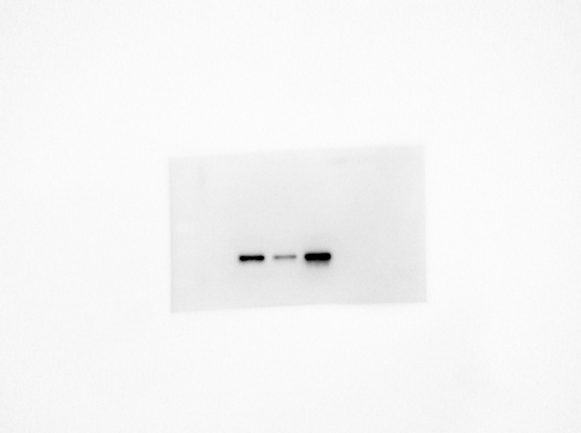

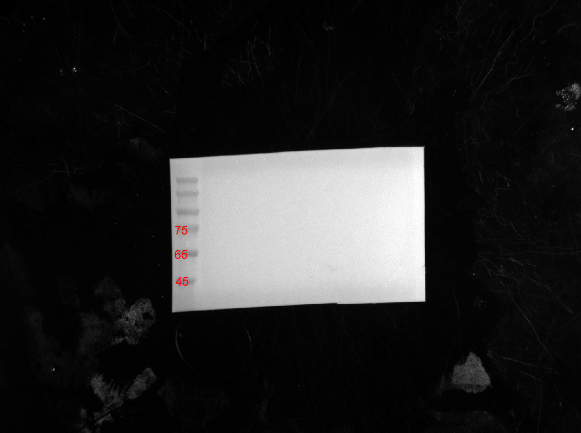


Figure 6D CYP27A1


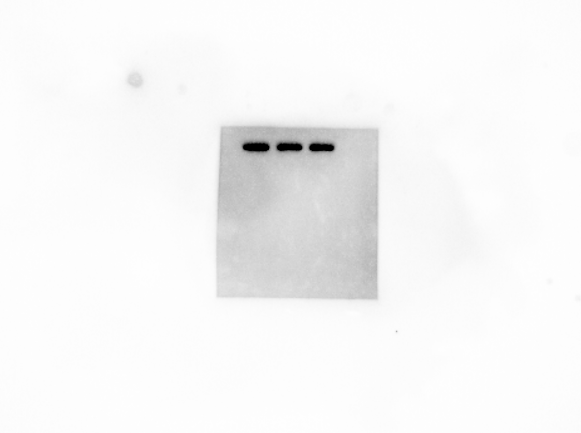

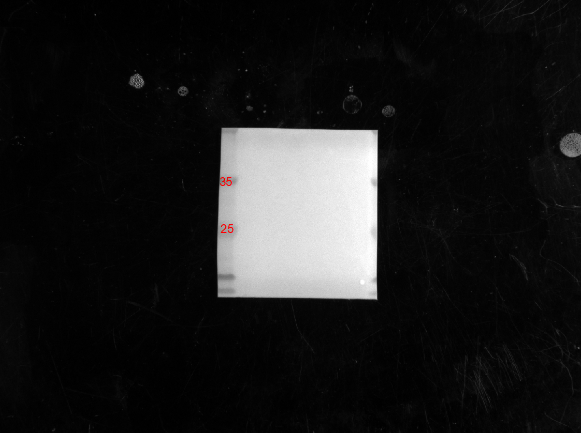


Figure 6D GAPDH


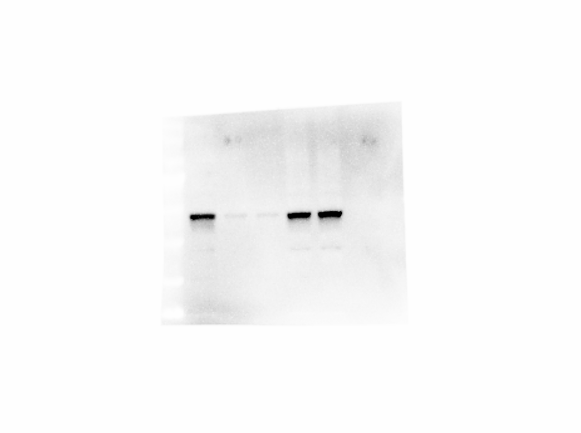

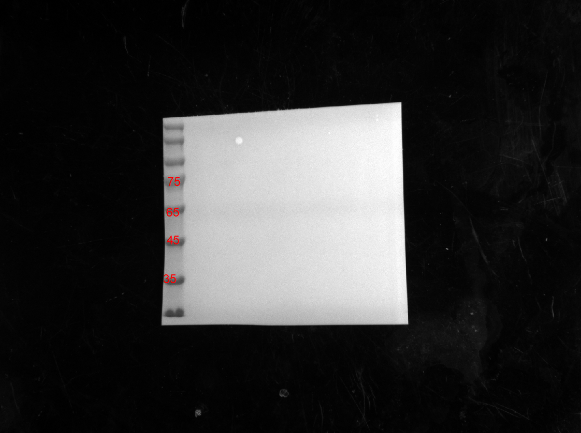


Figure 6E TUBB2B


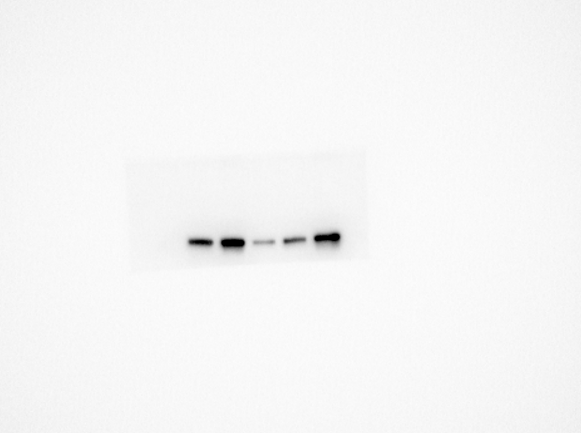

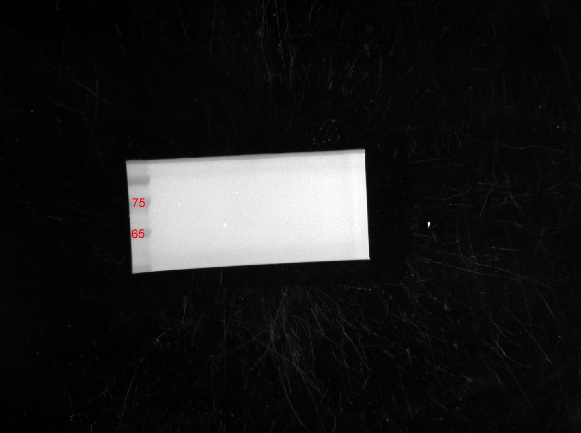


Figure 6E HNF4A


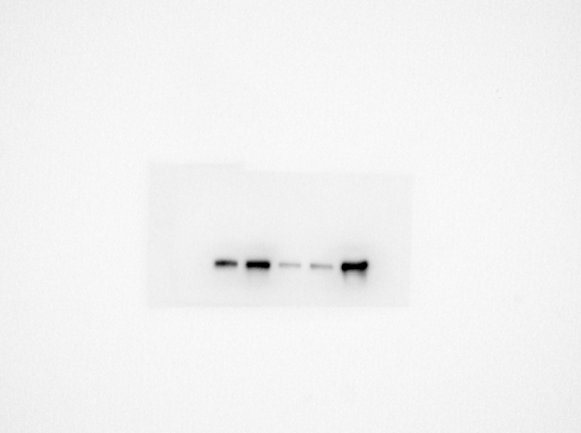

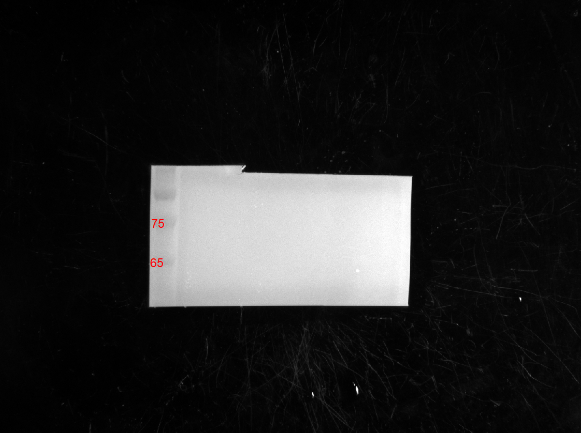


Figure 6E CYP27A1


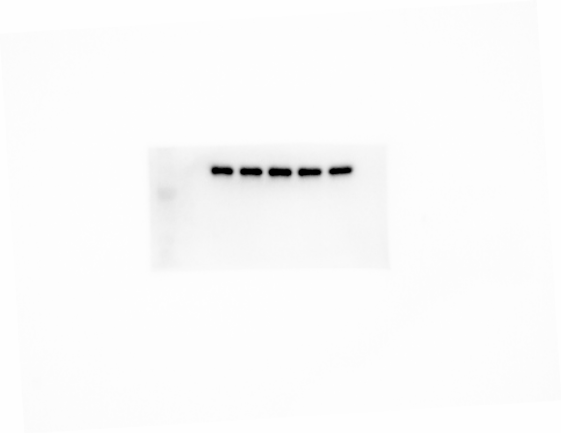

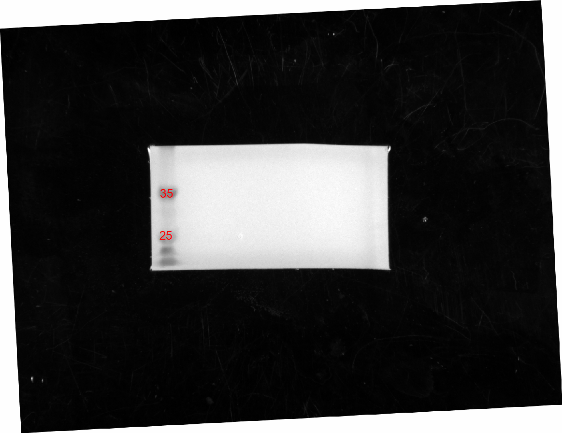


Figure 6E GAPDH


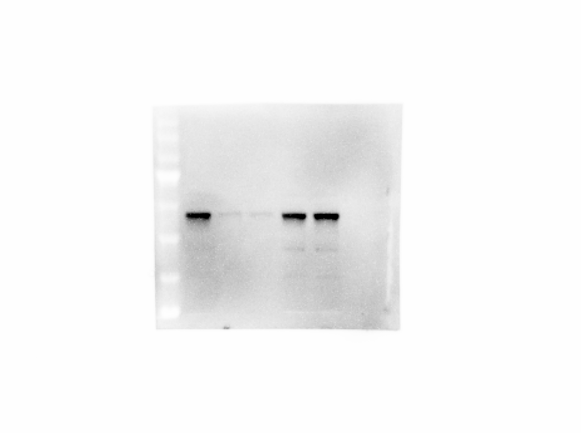

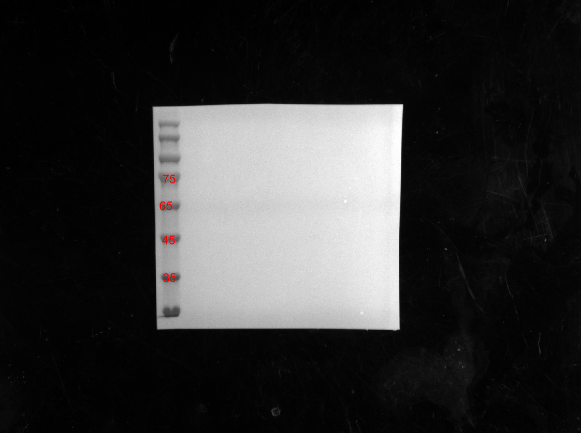


Figure 6F TUBB2B


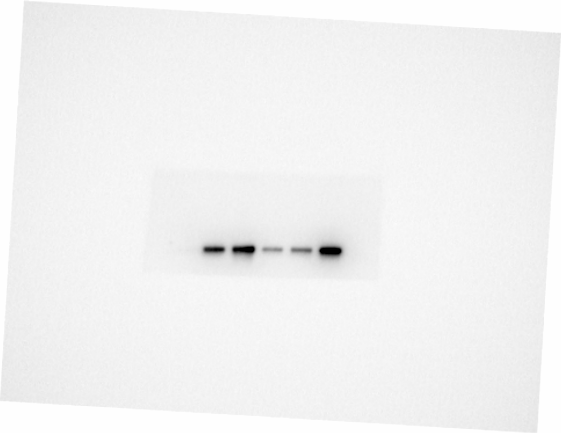

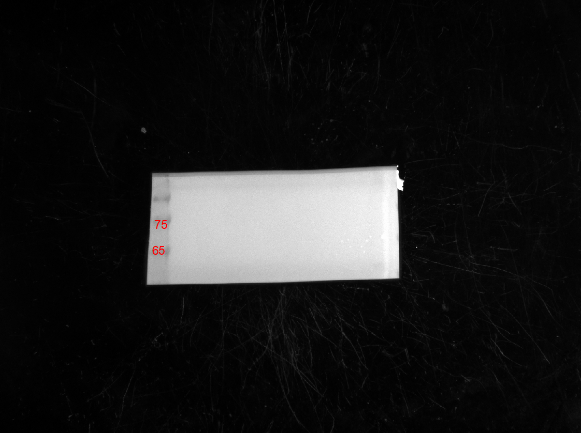


Figure 6F HNF4A

Figure 6F CYP27A1

Figure 6F GAPDH
